# Targeted delivery of Nitric Oxide triggered by α-Glucosidase to Ameliorate NSAIDs-induced Enteropathy

**DOI:** 10.1016/j.redox.2022.102590

**Published:** 2022-12-29

**Authors:** Xianglu Wang, Jiarui Shi, Zhixin Xu, Dan Wang, Yuguang Song, Guifang Han, Bangmao Wang, Hailong Cao, Yangping Liu, Jingli Hou

**Affiliations:** a The Province and Ministry Co-sponsored Collaborative Innovation Center for Medical Epigenetics, Tianjin Key Laboratory of Technologies Enabling Development of Clinical Therapeutics and Diagnostics, School of Pharmacy, Tianjin Medical University, Tianjin, 300070, China; b Department of Gastroenterology and Hepatology, General Hospital, Tianjin Medical University, Tianjin Institute of Digestive Diseases, Tianjin Key Laboratory of Digestive Diseases, Tianjin, China; c Department of Pathology, General Hospital, Tianjin Medical University, Tianjin, China

**Keywords:** Nitric oxide donor, α-Glucosidase, Small intestine-targeting, Small intestinal injury, Anti-inflammation

## Abstract

Nonsteroidal anti-inflammatory drugs (NSAIDs) increase risks of severe small intestinal injuries. Development of effective therapeutic strategies to overcome this issue remains challenging. Nitric oxide (NO) as a gaseous mediator plays a protective role in small intestinal injuries. However, small intestine-specific delivery systems for NO have not been reported yet. In this study, we reported a small intestine-targeted polymeric NO donor (CS–NO) which was synthesized by covalent grafting of α-glucosidase-activated NO donor onto chitosan. *In vitro* and *in vivo* experiments demonstrated that CS-NO could be activated by intestinal α-glucosidase to release NO in the small intestine. Pre-treatment of mice with CS-NO significantly alleviated small intestinal damage induced by indomethacin, as demonstrated by down-regulation of the levels of pro-inflammatory cytokines and chemokines CXCL1/KC. Moreover, CS-NO also attenuated indomethacin-induced gut barrier dysfunction as evidenced by up-regulation of the levels of tight junction proteins and restoration of the levels of goblet cells and MUC2 production. Meanwhile, CS-NO effectively restored the defense function of Paneth cells against pathogens in small intestine. Our present study paves the way to develop NO-based therapeutic strategy for NSAIDs-induced small intestinal injuries.

## Highlights

• A type of nitric oxide (NO) donor triggered by α-glucosidase to release NO was firstly developed.
• Polymeric NO donor (CS–NO) has been firstly developed by attaching α-glucosidase triggered NO-releasing donor to chitosan.
• NO release from CS-NO was studied by *in vivo* fluorescent imaging and electron paramagnetic resonance (EPR).
• NO delivery material (CS–NO) significantly alleviated indomethacin-induced small intestinal damage.

## Graphical abstract

A small intestine-specific polymeric NO donor (CS–NO) was developed by attaching a novel type of NO donor (α-glucosidase triggered NO donor) to chitosan for protection against NSAIDs-induced small intestinal injuries and inflammation.

**Figure:**
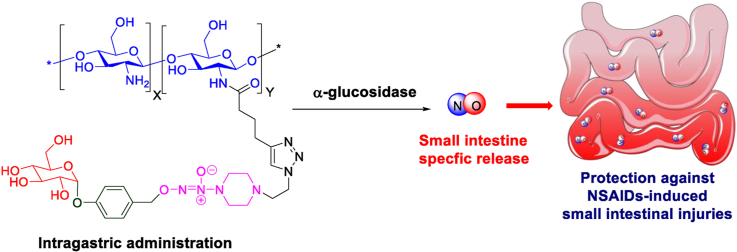

## 1 Introduction

Nonsteroidal anti-inflammatory drugs (NSAIDs) are the most widely used anti-inflammation drugs which can effectively reduce pain and inflammation. However, NSAIDs leads to significant damage in the gastrointestinal (GI) tract. While the damage to upper GI could be effectively healed by proton pump inhibitors, an increasing trend in lower GI events was observed among NSAID users [[1], [2], [3]]. Among these patients with NSAIDs-induced enteropathy, some of them are symptomatic or complicated ulcers that need therapeutic intervention [3]. Although much attention has been paid to NSAID-induced small bowel injuries, no effective therapeutic strategy has been developed [4].

Gaseous mediators including nitric oxide (NO), hydrogen sulphide(H_2_S) and carbon monoxide (CO), have beneficial effects on (GI) tract [5,6]. Among them, numerous studies evidenced that NO exerted a wide range of protective, reparative and anti-inflammatory effects in the GI tract, through increased intestinal microvascular blood flow and decreased leukocyte adhesion to the vasculature endothelium [7,8]. In addition, NO can also serve as a chain-breaking antioxidant to protect cells against lipid peroxidation [9,10]. NO, unlike the common compounds, has high reactivity and short biological half-life [11]. Thus, NO must be administered at the disease sites to improve its therapeutic outcome. To aim it, several local delivery systems for NO or its donors have been developed [[12], [13], [14], [15]]. Among them, the enzyme-prodrug strategy has accepted intense attention in which the biodistribution of the enzymes is utilized to precisely control the location of the NO release as well as its levels [[16], [17], [18]]. For example, enzyme-activatable NO donors have achieved liver- [19], prostate- [20] and reno-selective NO release [21]. In addition, glycosidase-triggered NO release from β-Gal-NONOates [17,22], and β-GlcNAc-NONOates [23] have also been reported in which the biocompatible sugars are used as the protecting groups. Despite these progresses, intestine-targeting delivery system for NO has not been reported so far.

α-glucosidase which catalyses the cleavage of α-1,4-glycosidic bond in oligosaccharides is prevalently located in the brush border of the enterocytes lining of intestine [24]. The specific distribution pattern of α-glucosidase could allow α-glycosylated drugs to achieve small intestine-specific release. Therefore, we firstly synthesized a water-soluble α-glycosylated NO donor , in which glucose as a triggering substrate is attached by an α-glycosidic bond to diazeniumdioate (NONOate) through a self-immolative linker (i.e., p-hydroxybenzyl). NONOates are chosen because this class of NO donors can spontaneously release NO under physiological conditions without changing the tissue redox status.

**Fig. 1:**
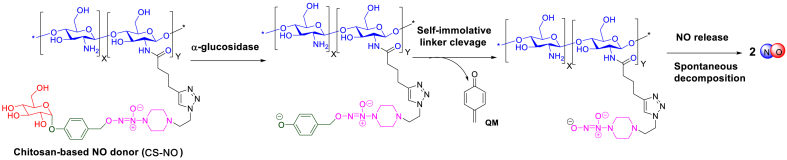
Alpha-glucosidase-induced NO-releasing mechanism from CS-NO.

The absorptions of chitosan in the GI tract were closely related to its molecular weight (MW): as the MW increased, the absorptions were decreased. High MW chitosan (230 KD) can be referred as an “inert” material because it is barely absorbed in the intestine [25].To prevent the intestinal absorption, the small molecular NO donor was covalently linked with high molecular weight (MW) chitosan [26] *via* amide bond to afford a chitosan-conjugated NO donor (named as CS-NO). We hypothesize that the small intestine-specific NO release from CS-NO can be achieved by the chitosan-promoted adherence to small intestine, followed by subsequent release of NO which is triggered by α-glucosidase in the intestine. Due to this unique property, the use of CS-NO enables protecting the inflamed intestine from pro-inflammatory challenges (Fig. 1).

## 2 Materials and methods

### 2.1 Chemicals and instruments

All solvents and reagents are commercially available and unless otherwise stated were used as received without prior purification. DCM was dried by distillation over CaH_2_. Methanol was dried over magnesium and distilled under an argon atmosphere. UV–Vis absorption spectra were taken on Hitachi U-3900 spectrophotometer. The^1^H NMR and^13^C NMR spectra were recorded on a BrukerAvance-400 FT nuclear magnetic resonance spectrometer. EPR spectra were recorded at room temperature (23 ± 1 °C) using a Bruker X-band EPR spectrometer. The following acquisition parameters were used: microwave power, 10 mW; modulation frequency, 100 kHz; modulation amplitude, 2 G. Fresh solution of Fe^2+^-(MGD)_2_ was prepared by mixing *N*-methyl-*d*-glucamine dithiocarbamate (MGD) sodium salt solution (50 mM in argon-purged double-distilled water) with ammonium ferrous sulphate hexahydrate solution (10 mM in argon-purged double-distilled water) under an argon atmosphere. NO concentration was calibrated using *2,2,6,6*-tetramethylpiperidin-1-yl)oxyl (TEMPO) as standard. Fluorescence imaging was performed on an IVIS® spectrum CT *in vivo* imaging system.

### 2.2 Animal preparation

8 weeks old male C57BL/6J mice, weighing 18–20 g, were purchased from HFK Bio (Beijing China). All mice were group-housed (5 mice per cage) under controlled temperature (22–23 °C) and photoperiod (12:12-h light-dark cycle) with chow food and water. All the experiments were carried out under the control of animal care and use committee in accordance with The Guidelines on Animal Experiments in Tianjin Medical University, Tianjin, China.

### 2.3 Synthesis of CS-NO

**Synthesis of compound 3:** Boron trifluoride-ether (20.3 mL, 138.6 mmol) was added dropwise into a cooled (0 °C) and stirred solution of peracetylated glucose (18 g, 46.2 mmol) and 4-methyl phenol (9.99 g, 92.4 mmol) in dry DCM (180 mL). The resulting mixture was gradually warmed to 50 °C and stirred for another 48 h. Then, the mixture was poured into ice water (200 mL) and extracted with DCM (3 × 300 mL). The combined organic phase was washed with saturated NaHCO_3_, water and brine, and then dried over Na_2_SO_4_. The solvent was evaporated under reduced pressure and the residue was purified by column chromatography (PE:EA = 4:1) to give the product 3 as a yellow syrup (7.0 g, 35%).^1^H NMR (400 MHz, CDCl_3_) δ 7.10 (d, *J* = 8.4 Hz, 2H), 6.97 (d, *J* = 8.4 Hz, 2H), 5.70 (t, *J* = 9.6 Hz, 1H), 5.68 (d, *J* = 3.7 Hz, 1H), 5.15 (t, *J* = 9.6 Hz, 1H), 5.03 (dd, *J* = 3.6 Hz, 10.2 Hz, 1H), 4.25 (dd, *J* = 4.4 Hz, 12.4 Hz, 1H), 4.16-4.12 (m, 1H), 4.05 (dd, *J* = 2.4 Hz, 12.4 Hz, 1H), 2.30 (s, 3H), 2.06-2.04 (m, 12H); ^13^C NMR (100 MHz, CDCl_3_) δ 170.5, 170.1, 169.6, 154.0, 132.5, 130.1, 116.5, 94.5, 70.5, 70.1, 68.4, 67.9, 61.6, 20.7, 20.64, 20.62, 20.60, 20.55 (one carbon less due to overlapping)

**Synthesis of compound 4:** To a solution of compound 3 (2.5 g, 5.71 mmol) in CCl_4_ (200 mL) was added *N*-bromo succinimide (1.1 g, 6.18 mmol) and benzyl peroxide (140 mg, 0.58 mmol). The resulting mixture was stirred at 80 °C for 5 h under an argon atmosphere. After cooling to room temperature, the precipitate was filtered and the solvent was evaporated under reduced pressure. The crude residue was purified by column chromatography (PE:EA = 3:1) to give the product 4 as a light yellow solid (2.0 g, 50%). ^1^H NMR (400 MHz, CDCl_3_) δ 7.34 (d, *J* = 8.4 Hz, 2H), 7.06 (d, *J* = 8.8 Hz, 2H), 5.74 (d, *J* = 3.6 Hz, 1H), 5.69 (t, *J* = 9.6 Hz, 1H), 5.15 (t, *J* = 9.6 Hz, 1H), 5.04 (dd, *J* = 10.4, 3.6 Hz, 1H), 4.48 (s, 2H), 4.24 (dd, *J* = 12.0, 4.4 Hz, 1H), 4.10-4.03 (m, 2H), 2.06-2.04 (m, 12H); ^13^C NMR (100 MHz, CDCl_3_) δ 170.6, 170.3, 169.7, 156.1, 132.7, 130.9, 116.9, 94.3, 70.5, 70.1, 68.4, 68.3, 61.7, 33.2, 20.8, 20.8, 20.8, 20.7 (one carbon less due to overlapping).

**Synthesis of compound 6:** Compound 5 (230 mg, 1.5 mmol), compound 4 (388 mg, 0.75 mmol) and KI (25 mg, 1.5 mmol) were added into a schlenk flask under an argon atmosphere, and then dry DMF (2 mL) was added into the reaction mixture at −2 °C. The reaction mixture was stirred for 24 h and water (20 mL) was added to quench the reaction. The mixture was extracted with EtOAc (3 × 20 mL). The combined organic phase was washed with brine and dried over Na_2_SO_4_. The solvent was evaporated under reduced pressure and the resulting residue was purified by column chromatography (PE:EA = 1:1) to afford compound 6 as a colourless syrup (300 mg, 70%).^1^H NMR (400 MHz, CDCl_3_) δ 7.33 (d, *J* = 8.4 Hz, 2H), 7.07 (d, *J* = 8.8 Hz, 2H), 5.73 (d, *J* = 3.6 Hz, 1H), 5.69 (t, *J* = 9.6 Hz, 1H), 5.16 (t, *J* = 9.6 Hz, 1H), 5.15 (s, 2H), 5.04 (dd, *J* = 10.4, 3.6 Hz, 1H), 4.25 (dd, *J* = 12.4, 4.4 Hz, 1H), 4.12-4.02 (m, 2H), 3.50-3.29 (m, 6H), 2.74-2.59 (m, 6H) 2.06-2.03 (m, 12H) (one active proton less); ^13^C NMR (100 MHz, CDCl_3_) δ 170.7, 170.3, 169.7, 156.4, 130.6, 130.4, 116.7, 94.3, 75.1, 70.5, 70.1, 68.4, 68.2, 61.6, 56.5, 51.7, 20.8, 20.8, 20.8, 20.7 (three carbon less due to overlapping).

**Synthesis of compound 7:** To a solution of compound 6 (140 mg) in anhydrous methanol (10 mL) was added a catalytic amount of MeONa. The resulting mixture was stirred at room temperature for 2 h. The solvent was evaporated under reduced pressure and the resulting residue was purified by column chromatography (DCM: methanol = 100:3) to afford the desirable compound 7 as a white foam (73 mg, 70%).^1^H NMR (400 MHz, D_2_O) δ 7.39 (d, *J* = 8.0 Hz, 2H), 7.15 (d, *J* = 8.4 Hz, 2H), 5.63 (d, *J* = 3.2 Hz, 1H), 5.23 (s, 2H), 3.91 (apparent t, *J* = 9.2 Hz, 1H), 3.72-3.68 (m, 4H), 3.52-3.44 (m, 3H), 3.38-3.33 (m, 4H), 2.65-2.61 (m, 4H), 2.57 (apparent t, *J* = 6.4 Hz, 2H); ^13^C NMR (100, D_2_O)δ 156.7, 130.9, 129.7, 117.2, 97.0, 75.7, 73.0, 72.5, 71.1, 69.3, 60.2, 55.1, 50.5, 50.3, 47.6.

**Synthesis of compound 8**：To a solution of compound 7 (175 mg, 362 μmol) and 5-hexynoicacid (45 mg, 398.14 μmol) in mixed solvent (*t*-BuOH: DCM: H_2_O = 1:1:1, 8 mL) were added CuSO_4_⋅5H_2_O (18 mg, 72.4 μmol) and sodium ascorbate (28 mg, 144.8 μmol). The reaction mixture was stirred at room temperature for 18 h. Then, the solvent was removed on vacuum to give the crude product, which was purified by C18-silica gel chromatography (from 20% to 30% MeOH in H_2_O) to give compound 8 as a white solid (140 mg, 65%). ^1^H NMR (400 MHz, MeOD) δ 7.81 (s, 1H), 7.34 (d, *J* = 8.4 Hz, 2H), 7.18 (d, *J* = 8.8 Hz, 2H), 5.50 (d, *J* = 3.6, 1H), 5.16 (s, 2H), 4.49 (t, *J* = 6.0 Hz, 2H), 3.85 (t, *J* = 9.2 Hz, 1H), 3.75-3.60 (m, 3H), 3.57 (dd, *J* = 9.6, 3.6 Hz, 1H), 3.44-3.35 (m, 5H), 2.86 (t, *J* = 6.0 Hz, 2H), 2.75 (t, *J* = 7.2 Hz, 2H), 2.66-2.63 (m, 4H), 2.34 (t, *J* = 6.8 Hz, 2H), 1.96 (quint, *J* = 6.8 Hz, 2H) (one active proton less); ^13^C NMR (100 MHz, D_2_O) δ 156.6, 131.0, 129.7, 117.2, 97.0, 75.9, 73.1, 72.6, 71.1, 69.4, 60.3, 55.4, 50.3, 49.1, 45.9, 24.8, 24.1 (four carbons less); HRMS (ESI+) *m*/*z* calculated for C_25_H_38_N_7_O_10_ [M+H]^+^: 596.2675; found 596.2678.

**Synthesis of CS-NO**：Chitosan (CS, MW = 20W, 0.5 g, 3.10 mmol) was dissolved in 2% HCl (20 mL, v/v) with stirring, then the solution was dialyzed (molecular weight cut off 8000) against distilled water for 4 days and lyophilized to give the lyophilized CS which was used in the following experiments. CS (0.5 g, 3.10 mmol) and compound 8 (250 mg, 0.419 mmol) was dissolved in water (25 mL), and then1-(3-dimethylaminopropyl)-3-ethylcarbodiimide hydrochloride (EDC.HCl, 323 mg, 1.68 mmol) and *N*-hydroxy succinimide (NHS, 193 mg, 1.68 mmol) were added in three batches at a time interval of 30 min. The resulting solution was stirred at room temperature for 24 h and dialyzed (molecular weight cut off 8000) against distilled water for 4 days and lyophilized to give CS-NO (580 mg) as a powder.

### 2.4 Analysis of the grafting density

Elemental analysis was used to quantitatively analyse the grafting density of NO-releasing compound 8 on CS-NO. The mass fraction of compound 8 in CS-NO (χ _(8)_) can be written as:

(1) MF_N(CS-NO)_ = (1-χ _(8)_) × MF_N(CS)_＋χ _(8)_ × MF_N(NO donor)_

That can be also written as:

(2) χ _(8)_ = (MF_N(CS-NO)_–MF_N(CS)_)／(MF_N(8)_–MF_N(CS)_)

The elemental analysis demonstrated that the mass fractions of nitrogen in CS-NO (MF_N(CS-NO)_) and CS (MF_N(CS)_) were9.00% and 7.98%, respectively, while the mass fraction of nitrogen in compound 8 (MF_N(8)_) was calculated to be 16.46%. Therefore, χ_(8)_ can be calculated to be 12.03%. The grafting density of compound 8 onto CS-NO can be calculated according to the following equation:

$${\%}_{\left(grafting\right)}=\frac{\left(12.03\%／M_{\left(8\right)}\right)}{\left(1-12.03\%\right)／M_{RSU}}$$

where M_(8)_ is the molar mass of compound 8 (595.6 g/mol), and M_RGU_ is the molar mass of one repeating sugar unit (161.2 g/mol). Thus, the grafting density of NO-releasing compound 8 on CS-NO was determined to be 3.7%.

### 2.5 Nanoparticle preparation and characterization

CS-NO nanoparticles were prepared by using tripolyphosphate (TPP) according to the ionotropic gelation method [27].The nanoparticles was obtained upon addition of an aqueous solution of TPP (2 mg/mL) to a CS-NO solution (4 mg/mL) in acetic acid solution(pH 3.5) under magnetic stirring. The particle size distribution, polydispersity index (PDI) and the morphology of the nanoparticles were evaluated by dynamic light scattering (DLS) and transmission electron microscopy (TEM).

### 2.6 *In situ* bioadhesion testing

The muco/bioadhesive properties make chitosan with long residence time in GI tract, thus improving absorption of the drug and increasing the drug's bioavailability. The bioadhesive property of polymer CS-NO was determined by an *in vitro* method evaluating the interaction of CS-NO with excised intestine [28]. Briefly, the jejunum was cut and washed with phosphate buffer (pH 6.0) until it was clean. Subsequently, 5 cm length jejunum was cut and fixed on a polyethylene support. 5 mg of polymer CS-NO was placed uniformly on the mucosa of the jejunum, which was then placed in a desicator maintained at > 80% relative humidity to allow CS-NO to hydrate and to interact with the mucosa. Finally, the mucosa of intestine was washed for 5 min with 0.1 N HCl and phosphate buffer (pH 6.0) respectively, at the rate of 22 mL/min using a peristaltic pump. After washing, the collected liquid was lyophilized to give the polymer CS-NO washed away. The percentage of CS-NO retained on the intestine was considered as an index of bioadhesion.

### 2.7 Detection of *invitro* and *in vivo* NO release from CS-NO

**Detection of NO release induced by α-glucosidase:** Griess reagent was firstly used to detect NO release from CS-NO. Briefly, CS-NO (0.5 mg/mL) was mixed with α-glucosidase (*EC 3.2.1.20, from S. cerevisiae*, 0.01 mg/mL) in phosphate buffer (50 mM, pH 5.0, 6.0 and 7.4). The resulting solution was incubated at 37 °C with gentle shaking. At designed time, 100 μL of the reaction mixture was taken out and mixed with 100 μL of 0.1% *N*-(1-naphthyl)-ethylene diamine dihydrochloride (Griess reagent A) and 100 μL of 1% sulphanilamide in 5% H_3_PO_4_(Griess reagent B). The resulting mixture was further incubated at room temperature for 30 min with periodic stirring. And the UV–vis absorbance intensity was measured at 548 nm. Then, the NO_2_^−^ concentrations at different time points were calculated on the basis of the standard curve in which the absorbance values were correlated with the known concentrations of NaNO_2_.

In addition to the Griess assay, EPR spin-trapping method was also used to directly detect the released NO. Briefly, 50 μL of CS-NO (1 mg/mL in water) was diluted with 30 μL of phosphate buffer (50 mM, pH 5.0, 6.0, 7.4), followed by addition of 10 μL of Fe^2+^-(MGD)_2_ (5 mM) and 10 μL of α-glucosidase (*EC 3.2.1.20, from S. cerevisiae*, 0.1 mg/mL in water). The final concentrations of CS-NO and α-glucosidase were 0.5 mg/mL and 0.01 mg/mL, respectively. The sample was quickly transferred into a EPR capillary (50 μL) and EPR spectra were recorded at different time points (15 min, 30 min, 1 h, 2 h, 3 h, 4 h and 6 h). Scan times, 10.

**Detect of the NO release in the presence of intestine homogenates:** Ileum was isolated from C57BL/6J mice and its homogenate was prepared using Potter-Elvejhem glass homogenizer. In brief, the intestine fraction was washed with water, dried with a paper towel and weighed. Then it was cut into several pieces and put into glass homogenizer. After addition of water (10 μL/mg), the tissue was grinded sufficiently to give the homogenates which were used immediately. To 70 μL of CS-NO solution in PBS (pH = 7.4, 20 mM, 5 mg/mL) were added 20 μL of Fe^2+^-(MGD)_2_ (5 mM) and 10 μL of fresh homogenates (10 mg/mL). The final concentrations of CS-NO and homogenates are 3.5 mg/mL and 1 mg/mL, respectively. The sample was quickly transferred into a 50 μL EPR capillary and EPR spectra were recorded at different time points (8 min, 15 min, 30 min, 1 h, 2 h, 3 h and 24 h). Scan times, 3.

**Detection of *In vivo* NO release:** To verify if NO can be released in small intestine after intragastric administration of CS-NO into mice, ferrous *N*-diethyl dithiocarbamate (Fe-DETC) was used as the spin-trapping reagent to detect NO release in the intestine. After intragastric administration of 50% CS-NO (weight of CS-NO/weight of CS = 1:1, 100 μL), DETC (500 mg/kg) and (NH_4_)_2_FeSO_4_·6H_2_O (35 mg/kg) as an iron citrate complex were injected separately subcutaneously into a mice with a time interval of 5 min [29]. After 15 min, 2 cm of duodenum and ileum were taken out and frozen in liquid nitrogen. Subsequently, the tissue was transferred to glass homogenizer and ethyl acetate (400 μL) was added. Then the tissue was homogenized, the supernatant was extracted and dried using nitrogen gas. Finally, 50 μL of ethyl acetate was added immediately and the sample was transferred to 50 μL capillary tube for EPR experiment. Scan times, 30.

Next, in *vivo* fluorescent imaging was also used to measure the NO release in mice. DAC-S, a water-soluble NO-sensitive fluorescent probe, was synthesized according to the previous literature [30]. C57BL/6J mouse was administered intragastrically with 100 μL of 50% CS-NO (20 mg/mL), followed by DAC-S (400 μM, 20 μL). The control mouse was administered intragastrically with 100 μL of CS (20 mg/mL) and DAC-S (400 μM, 20 μL). After 20 min, the mice were imaged with ICG filter pair on the IVIS Spectrum (PerkinElmer) to detect NO specific signal and total radiant efficiencies (p/s)/(μW/cm^2^) were measured.

### 2.8 The protective effect of CS-NO against the intestinal injury

**Experimental design:** To induce small intestinal injury, non-fasted animals were administered 20 mg/kg indomethacin in a 0.5% carboxymethylcellulose solution (4 mg/mL) once daily by gavage for two days. To examine the effect of CS-NO on NSAIDs-induced small intestinal injury, mice were randomly assigned into three groups: (1) PBS group (n = 8): phosphate buffered saline (PBS,100 μL) was administered to mice once daily via intragastric gavage for three days; (2) chitosan group (n = 7): mice were administered chitosan (100 μL, 20 mg/mL) once daily for three days; (3) CS-NO group (n = 10): 50% CS-NO (100 μL, 20 mg/mL) was administered to mice once daily for three days. Note that PBS, chitosan or 50% CS-NO was administered 1 h after indomethacin administration for the first two days. Mice were observed once daily for weight, and were sacrificed at the 4th day after indomethacin administration. The whole small intestine was taken out and examined for damage by the length of the small intestine and the status of macroscopically visible ulcers. Subsequently, the whole small intestine was divided into 3 segments of equal length. The distal small intestine was used for Hematoxylin and Erosin (H&E) staining, Periodic Acid Schiff (PAS) staining, immunohistochemical staining and PCR analysis.

**Histological assessment- H&E staining:** As previously reported, the whole small intestine was fixed in 10% buffered formalin, embedded in paraffin, and cut into 4-μm sections [31]. For evaluation of small intestinal injuries, sections [4-μm] were stained with H&E, and scored according to the reported histological damage scoring system [32] including the following six factors: epithelium, villus shape, villus tip, stroma, inflammation, and crypt status. Histological scores were determined blindly by two independent observers.Furthermore, Paneth cells can be identified by the presence of eosinophilic granules using H&E staining [33]. According to previous report [34], Paneth cells were divided into four categories by the patterns of lysozyme expression (represented in red, white represents areas that exclude lysozyme): normal (D0), disordered (D1), depleted (D2), and diffuse (D3). The percentage of total Paneth cells was also counted.

**PolyMerase Chain Reaction (PCR) analysis:** Total RNA was isolated from the distal small intestine tissue using the RNeasy Mini Kit (Qiagen)and reverse-transcribed to cDNA with TIAN Script RT Kit (TIANGEN, Inc). The gene expression analyses were quantified using quantitative real-time RT-PCR (StepOne Plus; Applied Biosystems, USA) and normalized with the glyceraldehyde-3-phosphate dehydrogenase (GAPDH) gene expression. The reactions were conducted by an initial incubation at 95 °C for 10 min, followed by 40 cycles of 95 °C for 15 s and 60 °C for 60 s. The expression levels of target gene mRNAs were calculated using the CT method (2^−ΔΔCT^). The primers for real-time RT-PCR are listed in Table S2.

**Periodic Acid Schiff (PAS) Staining:** Deparaffinized distal small intestine sections were incubated with 1% periodic acid solution (Sigma-Aldrich) for 10 min, then stained with Schiff's reagent (Sigma-Aldrich) for 40 min, and followed by counterstaining with hematoxylin solution for 2–5 min.

**Immunohistochemistry for MUC2:** The expression of mucin 2 (MUC2) in small intestinal were evaluated by immunohistochemistry. Deparaffinized distal small intestine sections were incubated with primary antibodies rabbit monoclonal anti-MUC2 (Santa Cruz Biotechnology, Inc) overnight at 4 °C, washed with PBS, and incubated with the biotinylated anti-rabbit secondary antibody (Santa Cruz Biotechnology, Inc) labelled with horseradish peroxidase (HRP) for 30 min at room temperature. Chromogenic development was performed using 3, 3′-diaminobenzidine, and the sections were then counterstained with hematoxylin. The expression of MUC2 was detected by calculating the absolute number of positively stained cells. Five random areas from a single section were viewed under fluorescence microscope (Leika, Germany) in at least 50 villi for each mouse.

**Statistical analysis:** Data were presented as the mean ± SEM. One-way ANOVA analysis was used to evaluate the statistical differences among different groups by using GraphPad Prism version 7.00. P < 0.05 was considered to be statistically significant.

## 3 Results and discussion

### 3.1 Synthesis and characterization of CS-NO

**Scheme 1:**
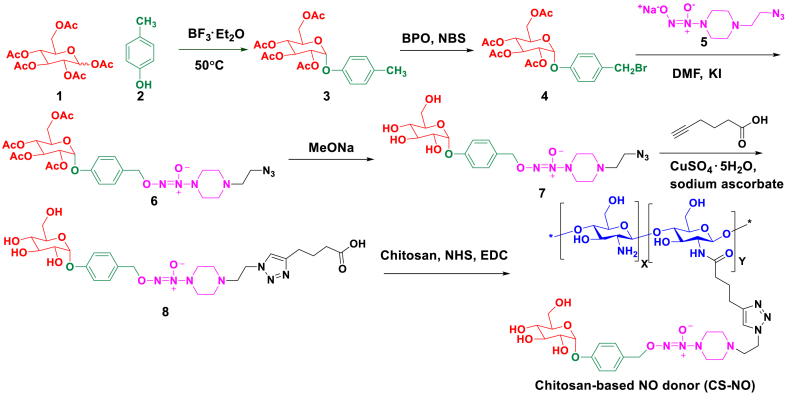
The synthetic route for preparation of CS-NO.

The chitosan-conjugated NO donor CS-NO was synthesized *via* six steps as shown in Scheme 1. Direct coupling of NONOate with the glucosyl residue in α-1,4 linkage is challenging due to the participatory effect of neighbouring group acyl at C2 [35], and the instability of the NONOate group towards metal or acids [36] which are usually used to catalyse the glycosylation. After several unsuccessful attempts, we chose an alternative strategy in which the glycosyl residue was conjugated with NONOates *via* a self-immolative linker (*p*-hydroxybenzyl). Peracetylated glucose (**1**) reacted with *p*-cresol (**2**) in the presence of boron trifluoride diethyl etherate to give the compound **3** which was assigned to be α anomer according to the relatively smaller coupling constant (^3^*J*_H1-H2_ = 3.7 Hz) calculated from its ^1^H NMR spectrum (Fig. S3) as compared to the reported value of ^3^*J*_H1-H2_ = 7–9 Hz for the corresponding β anomer [37]. Then, the α-anomer **3** was monobrominated using *N*-bromo succinimide (NBS) in the presence of benzoyl peroxide (BPO) as an initiator, followed by nucleophilic substitution with the NONOate (**5**) to afford the compound **6**. Thereafter, the compound **6** was deacetylated by catalytic amount of sodium methoxide (MeONa) to result in the compound **7** which was further coupled with 5-hexynoic acid *via* copper-catalysed alkyne-azide cycloaddition to afford compound **8.** In order to achieve specific distribution in intestine and lengthen the residence time of NO donor in GI, high MW chitosan (230 KD) was chosen as the scaffold to graft NO donor because it is barely absorbed in the GI tract, thus preventing CS-NO into systemic circulation and ensuring local release of NO in intestine [38]. As shown in Scheme 1, the compound **8** was covalently linked to chitosan *via* an amide condensation reaction to afford the desirable polymer **CS-NO**.

**Fig. 2:**
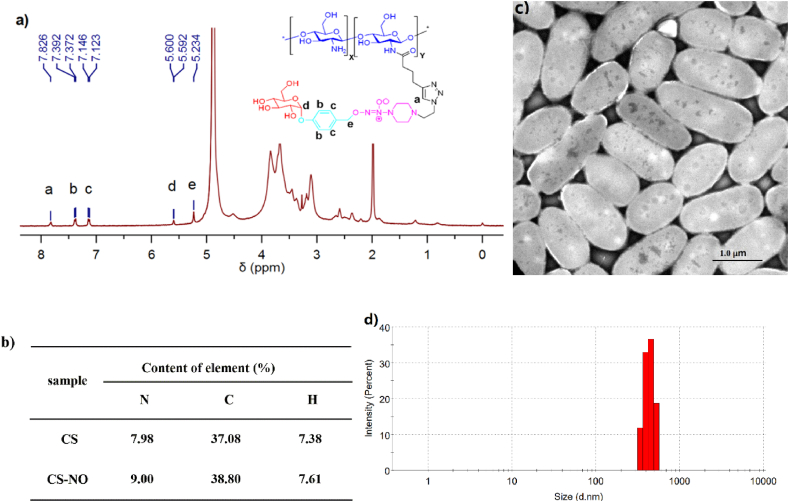
Characterization of synthesized CS-NO. (a) ^1^H NMR spectrum of CS-NO; (b) Elemental analysis of CS-NO; (c) TEM images of CS-NO nanoparticles. Scale bar: 1.0 μm; d) size distribution and hydrodynamic diameter from DLS.

CS-NO was characterized by ^1^H NMR and elemental analysis (Fig. 2a and b). In the ^1^H NMR spectrum of CS-NO measured in D_2_O (Fig. 2a), one singlet peak at 7.83 ppm was due to the proton on the triazole ring, and two doublet peaks at 7.38 and 7.13 ppm with coupling constants of 8.0 and 9.2 Hz, respectively, were attributed to the four protons on the phenyl ring. Additionally, the doublet peak at δ 5.60 p.m. with a coupling constant of 3.2 Hz was observed for the anomeric proton of the α-glucosyl moiety, and the singlet resonance at 5.23 p.m. corresponded to the two benzylic protons. Elemental analysis was performed to quantify the grafting density of the NO donor (i.e., the compound 8) onto the chitosan (Fig. 1). As shown in Fig. 2b, the mass fractions of nitrogen in CS-NO and CS are 9.00% and 7.98%, respectively. And the mass fraction of nitrogen in grafted NO donor (compound 8) was calculated to be 16.46% based on the molecular formula. From these data, the grafting density of compound 8 onto CS-NO can be calculated to be about 3.7% (see materials and method). Similar to chitosan, CS-NO could be prepared as nanoparticles by ionic gelation technique using TPP as cross-linking agent. The hydrodynamic diameter determined by DLS was ca. 440 nm with PDI of 0.21, indicating narrow distribution of the particle sizes. TEM images of CS-NO nanoparticles showed rod-like shape with a length of ca. 1 μm and a width of ca. 500 nm, which is different from DLS measurement. Subsequently, the bioadhesive property of CS-NO on excised intestinal tissue was evaluated using the method reported by P. Buri [39]. The binding efficiency of CS-NO reached to 60% which is comparable to chitosan with the binding efficiency as 70%. The good bioadhesive properties could result in long residence time in GI tract, which may improve NO release of CS-NO in the gut and increase its bioavailability.

### 3.2 *In vitro* NO release from CS-NO

To verify α-glucosyl capped NONOates could be activated by α-glucosidase to release NO, we firstly synthesized one similar α-glycosylated NO donor (α-Glc-NO,Scheme S1). *In vitro* experiments showed that α-Glc-NO was easily activated by α-glucosidase from yeast and in the intestinal homogenates of mice to release NO (Fig. S1 and Table S1). The NO release was substantially inhibited by acarbose (an α-glucosidase inhibitor), or completely inhibited by denatured α-glucosidase which was produced by heating α-glucosidase in boiling water for 5 min. In addition, α-Glc-NO showed good stability in aqueous HCl solution (pH 1) at 37 °C over 24 h. These results demonstrate that α-Glc-NO is responsive to α-glucosidase and can be used as the critical component for construction of the intestine-specific NO donor.

**Fig. 3:**
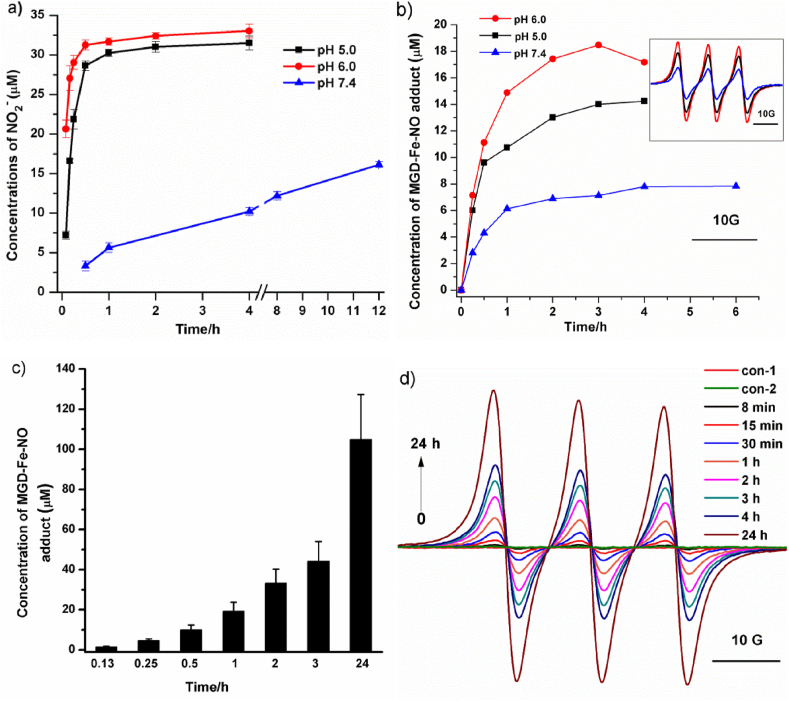
*In vitro* NO release from CS-NO. a) NO release from CS-NO (0.5 mg/mL) was measured by Griess assay in phosphate buffer solution (PB, pH 5.0, 6.0 and 7.4) containing α-glucosidase (0.01 mg/mL) at 37 °C; data was expressed at mean ± s.e.m, n = 3. b) The NO levels released from CS-NO (0.5 mg/mL)were measured by spin trapping technique in PB (pH 5.0, 6.0, 7.4) containing α-glucosidase(0.01 mg/mL); c) The NO levels released from CS-NO (3.5 mg/mL)were measured by spin trapping technique in PBS (pH 7.4, 20 mM) containing mice ileum homogenate (1 mg/mL); data was expressed at mean ± s.e.m, n = 3; d) EPR spectra of the NO adduct (i.e., MGD-Fe-NO) at different incubation times which were generated from the mixture of CS-NO (3.5 mg/mL), mice ileum homogenate (1 mg/mL) and MGD-Fe (1 mM) in PBS (pH 7.4, 20 mM).

Now that α-Glc-NO can be hydrolysed by the yeast α-glucosidase (Fig. S1 and Table S1), we then checked if the similar hydrolysis can occur for the polymeric CS-NO using Griess assay in which nitrite (NO_2_^−^), the final product of NO in air, is measured [40]. Fig. 3a shows the NO release from CS-NO (0.5 mg/mL) at different pHs (5.0, 6.0, and 7.4). Clearly, the NO release was relatively fast at low pHs (pH 5.0 and 6.0) and almost reached a plateau after 1-h incubation with the maximal NO concentration of about 32 μM. Comparatively, the NO release from CS-NO was much slower at the neutral pH but sustainable over 12 h. The pH-dependent release profile could be due to the protonation of the amine groups on the chitosan backbone at low pHs and the optimum condition (acid pH) for yeast α-glucosidase [41]. This protonation leads to a loose structure of CS-NO resulting from the strong electrostatic repulsive force between the adjacent sugar units. At pH 7, all of the chitosan chains were deprotonated and aggregated due to lack of electrostatic repulsion [42], resulting in much slower release of NO than that at pH 5–6. And, the majority of CS chains were still deprotonated and not fully extended as coil-like structure even at pH 5–6, thus causing the detected maximal NO concentration (∼32 μM) was much lower than theoretical value (∼180 μM) according to grafting density [42]. As such, the glucose residues on the CS-NO at low pHs are easily subject to hydrolysis by α-glucosidase. In addition, similar results were obtained in the presence of acarbose or denatured α-glucosidase (Fig. S2). All of the above results indicated the NO release from CS-NO was controlled by α-glucosidase.

To further confirm the NO release from CS-NO, electron paramagnetic resonance (EPR)-spin trapping experiments were also carried out using water soluble *N*-methyl-*d*-glucamine dithiocarbamate-iron (II) Fe^2+^(MGD)_2_ as a spin trap [43]. As shown in Fig. 3b, incubation of CS-NO (0.5 mg/mL) and Fe^2+^(MGD)_2_ (1 mM) with the yeast α-glucosidase at pH 5.0, 6.0 and 7.4 led to substantial amount of NO as evidenced by the appearance of a strong EPR triplet signal with a nitrogen hyperfine coupling constant of *a*_*N*_ = 12.7 G, which can be assigned to the complex of NO–Fe^2+^(MGD)_2_ (*a*_*N*_ = 12.5–13.2 G)[ [44] ,- [45]]. The results were similar to those of the Griess assay except for the lower accumulative concentration (∼18 μM) possibly due to the incomplete NO trapping.

To verify if CS-NO is capable of releasing NO in small intestine, EPR-spin trapping experiments were also carried out in the presence of homogenate of mice ileum. Mixture of the freshly prepared homogenate (1 mg/mL) with CS-NO (3.5 mg/mL) led to the triplet EPR signal of NO–Fe^2+^(MGD)_2_ which was enhanced with time. No signal was observed in the absence of CS-NO or the homogenate, indicating that CS-NO is triggered by the intestinal enzymes to release NO (Fig. 3c and d). The accumulative concentration of NO was about 105 μM after 24 h incubation with 3.5 mg/mL of CS-NO, consistent with the value of 18 μM from 0.5 mg/mL of CS-NO catalysed by the exogenous α-glucosidase (Fig. 3b). These results indicate that CS-NO can be activated by the enzymes located in the intestine to release NO.

It was worthy to note that an unsubstituted quinone methide (QM) was concomitantly released from CS-NO as one by-product (Fig. 1). This QM is a highly electrophilic reagent and could react with various nucleophiles such as GSH [46,47]. Potential depletion of GSH might compromise the beneficial effect from CS-NO [48,49]. However, QM also react with water to form *p*-hydroxybenzyl alcohol which play protective role against oxidative stress-related diseases [50,51]. Fortunately, CS-NO did show therapeutic effect on indomethacin-induced small intestinal injuries in mice.

### 3.3 Targeting delivery of NO to the intestine using CS-NO

**Fig. 4:**
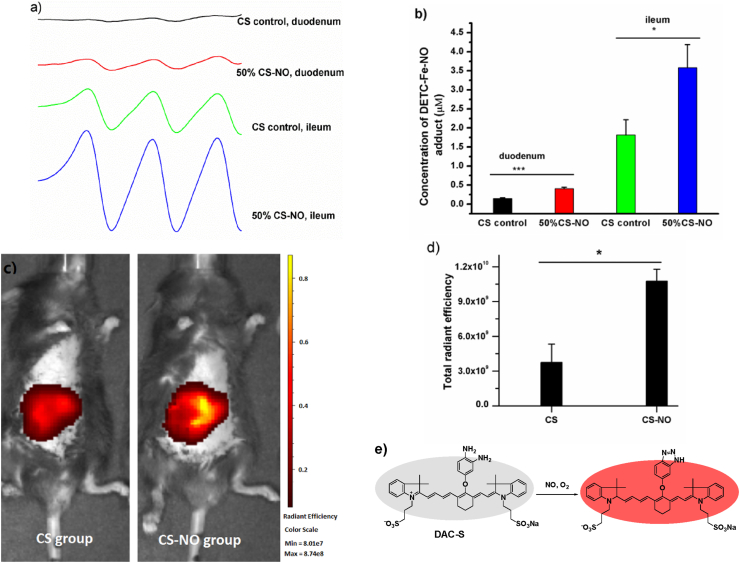
*In vitro* and *in vivo* NO release from CS-NO. a) EPR spectra showing the NO release from CS-NO in duodenum and ileum after intragastric administration of 100 μL of 50% CS-NO solution (20 mg/mL). b) The amount of the NO adducts (DETC)_2_Fe–NO) measured in duodenum and ileum; data was expressed at mean ± s.e.m, n = 5; *p < 0.05, ***P < 0.001. c) *In vivo* NO generation from CS-NO was detected by fluorescence imaging. CS group was administered intragastrically with 100 μL of 50% CS (20 mg/mL) and 20 μL of DAC-S (0.4 mM), while CS-NO group was administered with 100 μL of CS-NO (20 mg/mL) and DAC-S (0.4 mM, 20 μL). d) Total radiant efficiency from the mice administrated with CS and CS-NO respectively. Mean ± s.e.m; n = 3; *P < 0.05. Statistical analyses were performed with a two-tailed student's test. e) The sensing mechanism of DAC-S for NO.

Next, C57BL/6J mice were administered intragastrically with CS-NO solution, and NO generation in the intestine was evaluated by EPR assay using ferrous *N*-diethyl dithiocarbamate (Fe-DETC) as a spin trap (see materials and method). A characteristic triplet EPR signal (*a*_*N*_ = 12.78G) from the resultant NO adduct (DETC)_2_Fe–NO) was observed in both duodenum and ileum at room temperature (Fig. 4a). This signal was markedly elevated in the CS-NO group as compared to the CS control group, with ca. 2.8-fold and 2.0-fold enhancement respectively, indicating that the NO release mainly comes from CS-NO(Fig. 4b). Next, local release of NO in the intestine was also analysed by near-infrared (NIR) fluorescence imaging using the NO-sensitive probe DAC-S [30]. The probe is composed of two moieties with cyanine as the near-infrared (NIR) fluorophore and o-phenylenediamine as the recognition group. Under aerobic condition, *o*-phenylenediamine can react with NO to produce triazole, thus turning on the fluorescence of the probe DAC-S. The fast reaction rate between DAC-S and NO along with the NIR fluorescence allows for its use for *in vivo* imaging. Representative fluorescent images are shown in Fig. 4c. Compared to the mice administrated with CS, a significant fluorescence enhancement was observed in the mice administrated with CS-NO. Quantitative analysis of the fluorescence intensity showed that fluorescence signal was ca. 2.9-fold higher in CS-NO treated mice than CS treated mice (Fig. 4d). The above results indicate that NO could be released from CS-NO in small intestine, especially in ileum segment.

### 3.4 Suppression of indomethacin-induced body weight loss and intestinal injury by CS-NO

**Fig. 5:**
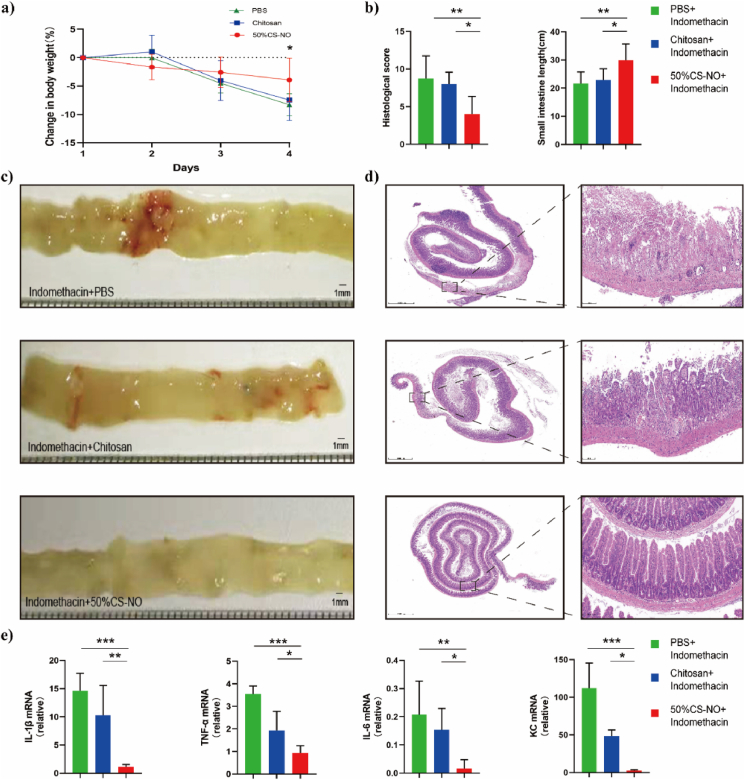
Suppression of indomethacin-induced body weight loss, intestinal injuries and the intestinal low-grade inflammation in mice by CS-NO. a) Time-course percentage changes of body weights after indomethacin administration. b) Histological scores from the small intestine (Left) and the complete length of small intestine (Right). c) Mucosal bleeding of the distal small intestine. d) H&E staining of the distal small intestine. Scale bars: 1000 μm (Left), 100 μm (Right). e) Expression of inflammatory cytokines including TNF-α, IL-1β, IL-6 and KC were quantitatively analysed by Realtime PCR. Total RNA was extracted from the distal small intestine. CS-NO: n = 10, Chitosan: n = 7, PBS: n = 8. *p < 0.05, **p < 0.01, ***p < 0.001.

Since NO has been shown to protect against the intestinal injuries induced by NSAIDs [11], the therapeutic effect of CS-NO on indomethacin-induced small intestinal injuries in mice was examined. To exclude the effect of chitosan (CS) backbone, CS was also used as a control group in addition to the PBS group. The small intestinal injury was assessed by body weight change, the area of macroscopically visible lesions and change of the intestinal length. As shown in Fig. 5a, indomethacin caused notable weight loss, and CS-NO significantly alleviated weight loss. No significant difference in the changes of body weight was observed between PBS-treated and chitosan-treated groups, indicating that CS has negligible therapeutic effect. CS-NO obviously attenuated the indomethacin-induced intestinal length reduction (Fig. 5c) and the area of macroscophic visible lesions (Fig. 5c). Histological analysis revealed destruction of the upper part of the epithelium, crypt loss and inflammatory infiltration into the lamina propria, submucosa, and serosa in control groups, and CS-NO resulted in more intact surface epithelium and crypt glands than those in control groups (Fig. 5d). CS-NO attenuated the intestinal damage as indicated by histological scores which were evaluated by inflammation severity, the extent of injury and crypt damage (Fig. 5b). The histological damage scores of CS–NO–treated mice were significantly lower than those of control groups (Fig. 5b).

Since the scores from histopathological examinations were very different between the CS-treated and control groups, we further investigated the expression of proinflammatory cytokines and chemokines in these small intestine samples. Interleukin-1β (IL-1β), Tumour necrosis factor-α (TNF-α), Interleukin-6 (IL-6), and Chemokine Ligand 1 (CXCL1/KC) were significantly lower in the CS-NO groups than in control groups (Fig. 5e), while the PBS control group had relatively higher levels of the above proteins than the CS control group, possibly due to the anti-inflammatory effect of chitosan. These findings suggested that CS-NO protected the small intestine against the indomethacin-induced damage.

### 3.5 Protection of the intestinal barrier against indomethacin by CS-NO

**Fig. 6:**
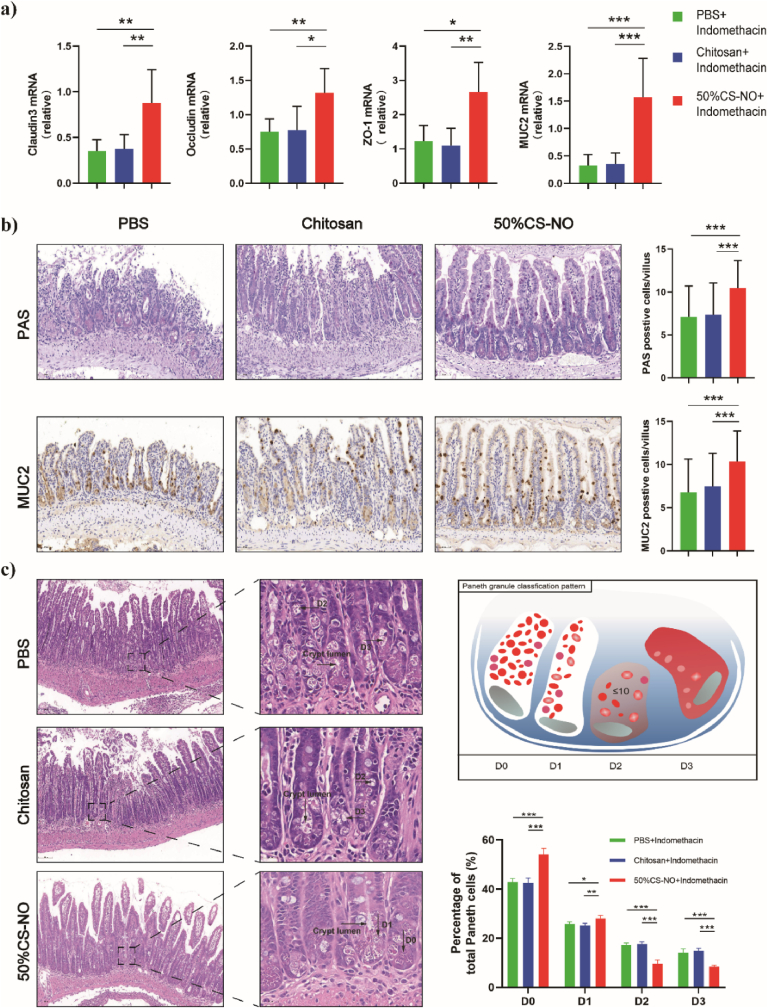
Protection of the intestinal barrier against indomethacin-induced small intestinal injury by CS-NO. a) The relative expressions of the intestinal tight junction proteins including Claudin3, Occludin, ZO-1, and Goblet cell product MUC2 were measured by Realtime PCR. Total RNA was extracted from the distal small intestine. b) Periodic acid Schiff staining for Goblet cells and MUC2 staining in the distal small intestine (Left) and the number of positive staining cells in each villus (Right). Scale bars: 50 μm. c) The morphology of Paneth granules was presented by H&E staining. The morphology of Paneth granules could be classified into four categories by the expression of lysozyme (represented in red, white represents areas that exclude lysozyme): normal (D0), disordered (D1), depleted (D2), and diffuse (D3). Percentage of Paneth cells showed normal (D0) and abnormal (D1-D3) patterns of lysozyme expression. One hundred Paneth cells for each mouse were scored. Scale bars: 100 μm (Left), 20 μm (Right).CS-NO: n = 10, Chitosan: n = 7, PBS: n = 8. *p < 0.05, **p < 0.01, ***p < 0.001. (For interpretation of the references to colour in this figure legend, the reader is referred to the Web version of this article.)

Growing evidence reveals that the destruction of the gut barrier is closely associated with GI disorders such as inflammatory bowel disease and colorectal cancer [[52], [53], [54]]. The tight junction proteins including zonula occludens-1 (ZO-1), Claudin-3, and Occludin are considered as the most important factors in maintaining the integrity of the gut barrier. Therefore, we studied the effect of CS-NO on the tight junction proteins in indomethacin-treated mice. Realtime PCR results (Fig. 6a) showed that the indomethacin treatment significantly reduced the expressions of Claudin 3, ZO-1, and Occludin (see control groups), while these proteins were notably up-regulated after administration of CS-NO to the indomethacin-treated mice. No statistical difference was observed between the PBS group and chitosan group. Moreover, the levels of MUC2 were also measured which is one of the most important products secreted from goblet cells and participates in the formation of mucous layer. In our studies, goblet cells and MUC2 in each villus were decreased in the PBS and chitosan groups, while CS-NO restored the quantity of goblet cells and MUC2 (Fig. 6b), as shown by PAS staining and immunohistochemical staining.

In addition, Paneth cells, characteristic cells of the small intestinal glands, are primarily located at the base of crypts in the small intestine and secrete the defensins and lysozyme in response to bacterial, cholinergic and other stimuli to maintain the gastrointestinal barrier [55,56]. We evaluated the morphology of Paneth cell by lysozyme expression which normally presents as granular form in H&E staining [33]. Compared with PBS and chitosan groups, CS-NO group displayed a significant increase in normal expression pattern (D0) of Paneth cells and an obvious decrease in abnormal expression pattern (D1-D3) of Paneth cells (Fig. 6c). Hence, CS-NO increased the normal Paneth cells ratio and recovered the defense function of Paneth cell against pathogens. Taken together, our data suggested that CS-NO significantly alleviated the destruction of the gut barrier in the indomethacin-treated mice.

## 4 Conclusion

In summary, we report the first synthesis of α-glucosidase-activatable polymeric NO donor (CS–NO). The grafting of the α-glucosidase-activatable NO-releasing moiety onto the high MW chitosan not only extends the residence time of CS-NO in the GI tract but also avoids its systemic circulation, thus enabling the small intestine-specific NO release. Owing to its unique property, CS-NO effectively attenuates indomethacin-induced small intestinal injuries in mice by alleviating the intestinal inflammation and ameliorating the gut barrier. Therefore, such type of NO donors shows great promise for NSAIDs-induced small intestinal injuries. Moreover, our present study provides a new example for development of enzyme-triggered tissue-targeting NO donors [12,[16], [17], [18]].

## Author contributions

Jingli Hou, Yangping Liu, Hailong Cao and Bangmao Wang conceived the original concept and supervised this project. JingliHou and Jiarui Shi synthesized all the compounds and carried out the NO-release experiment; Xianglu Wang and Dan Wang performed the *in vivo* evaluation; Zhixin Xu, Guifang Han and Yuguang Song help to measure the NO generation. All authors wrote the paper.

## Declaration of competing interest

There are no conflicts to declare.

## Data Availability

Data will be made available on request.
