# Supplementary material for: Targeted delivery of Nitric Oxide triggered by α-Glucosidase to Ameliorate NSAIDs-induced Enteropathy
Source: Redox Biol. 2022 Dec 29;59:102590. doi: 10.1016/j.redox.2022.102590 (PMC9813757; doi:10.1016/j.redox.2022.102590)
Supplement: Multimedia component 1 [file mmc1.docx]

**Supporting Information**

Targeted delivery of Nitric Oxide triggered by α-Glucosidase to Ameliorate NSAIDs-induced Enteropathy

Xianglu Wang,^a,b,#^ Jiarui Shi,^a,#^ Zhixin Xu,^a^ Dan Wang,^c^ Yuguang Song,^a^ Guifang Han,^a^ Bangmao Wang,^b*^ Hailong Cao,^b*^ Yangping Liu,^a*^ Jingli Hou^a*^

1. The province and ministry co-sponsored collaborative innovation center for medical epigenetics, Tianjin Key Laboratory of Technologies Enabling Development of Clinical Therapeutics and Diagnostics, School of Pharmacy, Tianjin Medical University, Tianjin 300070, P. R. China
2. Department of Gastroenterology and Hepatology, General Hospital, Tianjin Medical University, Tianjin Institute of Digestive Diseases, Tianjin Key Laboratory of Digestive Diseases, Tianjin, China
3. Department of Pathology, General Hospital, Tianjin Medical University, Tianjin, China

#. These authors contributed equally to this work

* Corresponding authors

E-mail addresses: [mwang02@tmu.edu.cn](mailto:mwang02@tmu.edu.cn) (B. Wang); [caohailong@tmu.edu.cn](mailto:caohailong@tmu.edu.cn) (H. Cao); [liuyangping@tmu.edu.cn](mailto:liuyangping@tmu.edu.cn) (Y. Liu); [houjingli@tmu.edu.cn](mailto:houjingli@tmu.edu.cn) (J. Hou)

**List of Contents**

1. Synthesis of compound α-Glc-NO……………………………………………………………………………………….2
2. Synthesis of Probe DAC-S……………………………………………………………………………………………………2
3. NO release from compound α-Glc-NO……………………………………………………………………………….3

4. NO release from CS-NO………………………………………………………………………………………………………5

[5. PolyMerase Chain Reaction (PCR) analysis………………………………………………………………………….6](#_Toc527464765)

[6. NMR spectra of all compounds……………………………………………………………………………………………7](#_Toc527464766)

1. **Synthesis of compound** α**-Glc-NO**

Scheme s1: The synthetic route of α-Glc-NO

**Compound 6-a**

Compound 5-a (PYRRO/NO, 230 mg, 1.5 mmol), compound 4 (388 mg, 0.75 mmol) and KI (25 mg, 1.5 mmol) were added into schlenk flask under an argon atmosphere, then dry DMF (2 mL) was added into the reaction mixture at -2°C and stirred for 24 h. After the reaction was completed, water was added to quench the reaction. The mixture was extracted with EtOAc three times and the combined organic phase was washed with brine, dried over Na_2_SO_4_. The solvent was evaporated under reduced pressure to give a crude compound, which was purified by column chromatography to afford compound 4a as colorless syrup (280 mg, 65%). ^1^H NMR (400 MHz, CDCl_3_) δ 7.35 (d, *J* = 8.6 Hz, 2H), 7.07 (d, *J* = 8.6 Hz, 2H), 5.74 (d, *J* = 3.6 Hz, 1H), 5.70 (d, *J* = 9.7 Hz, 1H), 5.16 (t, *J* = 9.6 Hz, 1H), 5.12 (s, 2H), 5.04 (dd, *J_1_* = 3.6 Hz, *J_2_* = 10.2 Hz, 1H), 4.26 (dd, *J_1_* = 4.4 Hz, *J_2_* = 12.3 Hz, 1H), 4.13-4.08 (m, 1H), 4.05 (dd, *J_1_* = 2.1 Hz, *J_2_* = 12.3 Hz, 1H), 3.53-3.49 (m, 4H), 2.07-2.04(m, 12H), 1.95-1.91 (m, 4H); ^13^C NMR (100 MHz, CDCl_3_) δ 170.6, 170.2, 169.6, 156.2, 130.7, 130.5, 116.5, 94.2, 74.6, 70.4, 70.0, 68.2, 68.0, 61.5, 50.9, 22.8, 20.73, 20.67, 20.62

**Compound α-Glc-NO**

To the solution of compound 6-a (140 mg) in anhydrous methanol (20 mL) was added a catalytic amount of MeONa. The resulting mixture was stirred at room temperature for 2 h. Concentration of the solvent followed by column chromatography gave the desirable compound α-Glc-NO as white solid (40 mg, 40%). ^1^H NMR (400 MHz, MeOD) δ 7.34 (d, *J* = 8.8 Hz, 2H), 7.17 (d, *J* = 8.4 Hz, 2H), 5.49 (d, *J* = 3.6 Hz, 1H), 5.11 (s, 2H), 3.85 (t, *J* = 9.2 Hz, 1H), 3.75-3.60 (m, 3H), 3.56 (dd, *J_1_* = 3.6 Hz, *J_2_* = 9.6 Hz, 1H), 3.49-3.45 (m, 4H), 3.42 (t, *J* = 9.2 Hz, 1H), 1.94-1.90 (m, 4H); ^13^C NMR (100 MHz, MeOD) δ 158.9, 131.5, 131.3, 118.1, 99.2, 76.0, 74.9, 74.5, 73.3, 71.5, 62.4, 52.0, 23.7

1. **Synthesis of Compound DAC-S**

Scheme s2: The synthetic route of DAC-S

**Compound IR-NO_2_**

To the anhydrous DMF solution (6 mL) was added 4-Amino-3-nitrophenol (100 mg, 0.65 mmol) and NaH (30 mg, 0.65 mmol, 60% in mineral oil). The mixture was stirred at room temperature for 20 min under an argon atmosphere. Then a solution of IR-783 (100 mg, 0.13 mmol) in anhydrous DMF (4 mL) was added to the reaction via a syringe. The reaction mixture was further stirred for 6 h. After removal of the DMF under reduced pressure, the crude product was purified by silica gel chromatography with 30% CH_3_OH in DCM to afford the intermediate (IR-NO_2_) as a dark green solid (90 mg, 80%). ^1^HNMR (400 MHz, CD_3_OD) δ 7.98 (d, 2H, *J* = 14.0 Hz), 7.71 (d, 1H, *J* = 2.8 Hz), 7.18-7.40 (m, 9H), 7.07 (d, 1H, *J* = 9.2 Hz), 6.22 (d, 2H, *J* = 14.0 Hz), 4.14 (t, 4H, *J* = 6.8 Hz), 2.87 (t, 4H, *J* = 6.8 Hz), 2.77 (t, 4H, *J* = 6.0 Hz), 1.91-2.05 (m, 10H), 1.42 (s, 12H).

**Compound DAC-S**

To a solution of IR-NO_2_ (50 mg, 0.06 mmol) in MeOH (2 mL) and concentrated HCl (0.3 mL) was added SnCl_2_-2H_2_O (200 mg, 0.9 mmol). The solution was stirred at room temperature for 6 h under an argon atmosphere. The precipitate was removed by filtration and the filtrate was concentrated under reduced pressure. Then the crude product was purified by silica gel chromatography with 35% CH_3_OH in DCM to afford the desired product as a dark green solid (20 mg, 40%). ^1^HNMR (400 MHz, CD_3_OD) δ 8.07 (d, 2H, *J* = 14.4 Hz), 7.16-7.37 (m, 8H), 6.68 (d, 1H, *J* = 8.4 Hz), 6.55 (d, 1H, *J* = 2.4 Hz), 6.30 (dd, 1H, *J* = 8.4, 2.8 Hz), 6.15 (d, 2H, *J* = 14.0 Hz), 4.11 (t, 4H, *J* = 6.8 Hz), 2.87 (t, 4H, *J* = 6.4 Hz), 2.73 (t, 4H, *J* = 5.6 Hz), 1.92-2.03(m, 10H), 1.40 (s, 12H).

1. **NO release from compound α-Glc-NO**

**Method A:** The decomposition of functional group NONOate was determined by UV-Vis spectrophotometry (Hitachi, U-3900). α-Glc-NO was dissolved in water to make 10 mM stock solution. α-glucosidase (*EC 3.2.1.20, from* S. cerevisiae*, sigma*) was dissolved in 30% (v/v) glycerol in deionised water to prepare 2 mg/mL stock solution, which were diluted 10 times to 0.2 mg/mL as working solution with phosphate buffer (PB, 50 mM, pH 7.4). The decomposition of the NONOate group was measured about 11 min with a cycle time of 1 min. The measurements were taken against a blank containing the phosphate buffer and were performed at following condition: α-Glc-NO (50 µM) and α-glucosidase (0.01 mg/mL).

**Method B:** The total NO amount was determined by nitrite measurement. Dilute 5 µL solution of α-Glc-NO (1 mM) to 90 µL using PB buffer (50 mM, pH 7.4). Then 10 µL of 0.1 mg/mL α-glucosidase (*EC 3.2.1.20, from* S. cerevisiae) was added to the substrate and the reaction mixture was incubated for an hour. Next, the solution was diluted 6 times with PB buffer (50 mM, pH 7.4) and 100 μL of the resulting solution was taken out to mix with 100 µL of 0.1% *N*-(1-naphthyl)-ethylenediamine dihydrochloride and 100µL of 1% sulphanilamide in 5% H_3_PO_4_. The resulting mixture was incubated for 30 min with periodic stirring and the absorbance of the solution was measured at 548 nm. The same method was used for an aqueous solution (pH = 1, HCl) of α-Glc-NO. The relationship of absorbance and concentrations of nitrite was constructed by drawing a standard curve from known concentrations of NaNO_2_.

**Method C:** The NO release was determined by EPR spectroscopy. All EPR spectra were recorded at room temperature using a Bruker X-band EPR spectrometer. The following acquisition parameters were used: microwave power, 10 mW; modulation frequency, 100 kHz; modulation amplitude, 2.0 G. The solution of Fe^2+^-(MGD)_2_ (5 mM) was prepared by mixing *N*-methyl-*D*-glucamine dithiocarbamate (MGD) sodium salt solution (50 mM in argon-purged double-distilled water) with ammonium ferrous sulphate hexahydrate solution (10 mM in argon-purged double-distilled water) in a ratio of 1:1 (v/v) under an argon atmosphere. NO concentration was calibrated using [2,2,6,6-tetramethylpiperidin-1-yl)oxyl](http://www.lskhsw.com/product/2.html) (TEMPO) as standard.


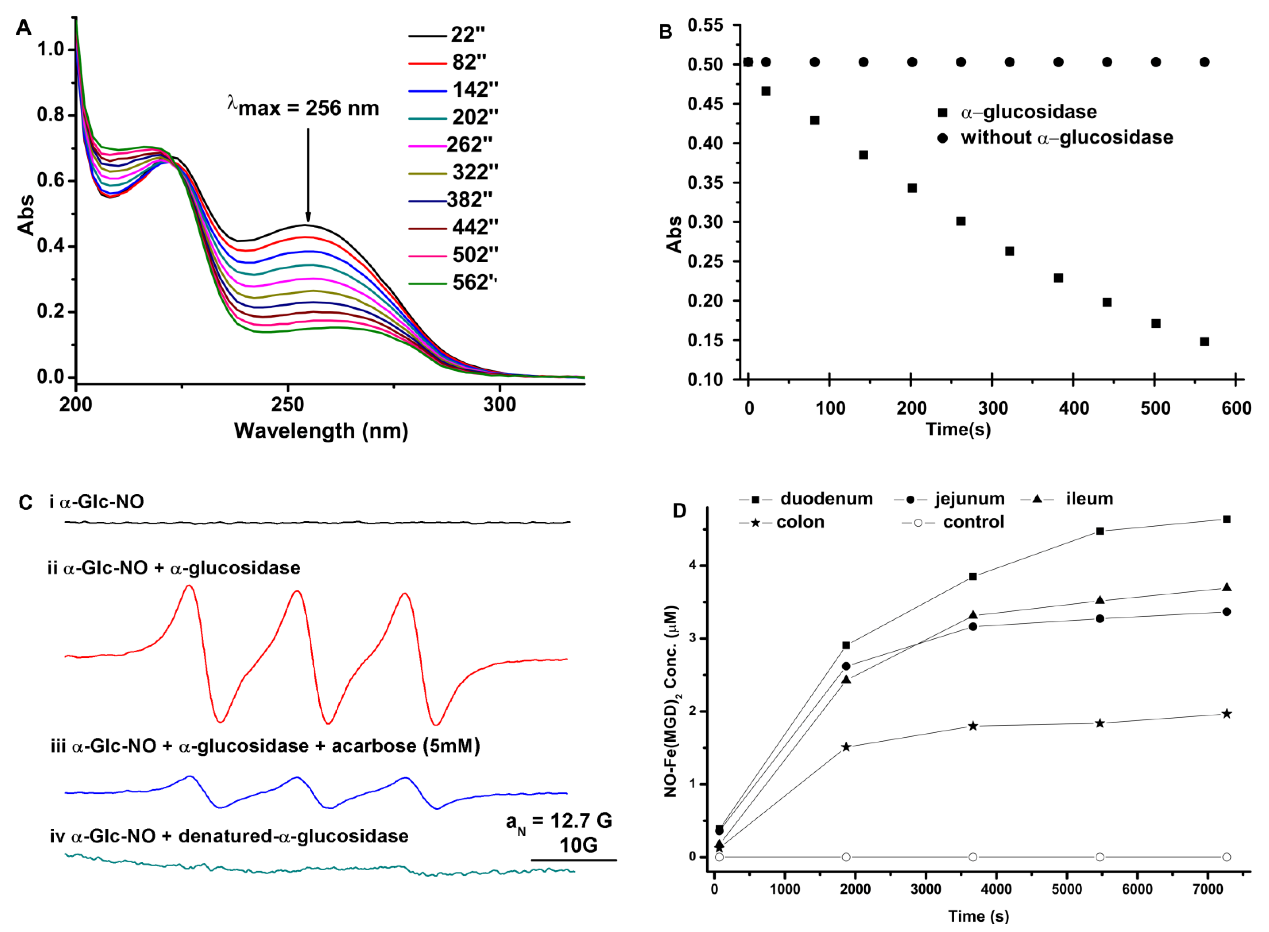


Figure s1. (A) UV-Vis spectral changes with time for the solution of α-Glc-NO (50μM)in PB (50 mM, pH 7.4) in the presence of α-glucosidase (0.01 mg/mL); (B) plot of UV-Vis absorbance intensities at 256 nm as a function of time for the solution of α-Glc-NO(50 μM) in PB (50 mM,pH 7.4) with () or without () of α-glucosidase (0.01 mg/mL). (C) EPR spectra obtained after 1h-incubation of Fe^2+^-(MGD)_2_ (1mM) with α-Glc-NO (50 μM) in the absence (i) and presence of (ii) α-glucosidase (0.01 mg/mL) or (iv) heat-denatured-α-glucosidase; (iii) the same as (ii) but in the presence of acarbose (5 mM). (D) Time course of the increase in EPR signal intensity of NO-Fe(MGD)_2_ in different fractions of intestinal homogenate (0.1mg/mL) for α-Glc-NO (2 μM) in PBS buffer (pH 7.4). The EPR intensities were calibrated into concentrations of the NO-Fe(MGD)_2_ using TEMPO as a standard.

Table s1. Formation of nitrite (NO_2_^-^) from α-Glc-NO measured by the Griess method


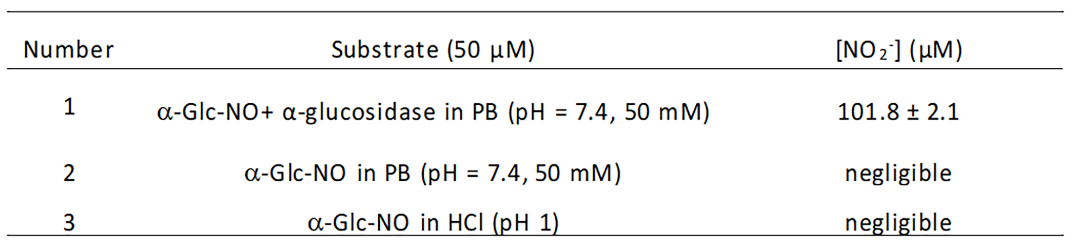


Note:(1) The solution ofα-glucosidase (0.01 mg/mL) with α-Glc-NO (50µM) in PB (pH = 7.4, 50 mM) was incubated at 37 °C for 1 hourwith shaking at 300 rpm. (2 and 3) The solution of α-Glc-NO (50µM) in PB (pH = 7.4, 50 mM) or HCl solution (pH =1) was incubated at 37 °C for 1 hwith shaking at 300 rpm.Then Griess reagent A and B were added in order andthe UV-Vis absorbance at 540 nmwas measured.

1. **NO release from CS-NO**

The NO release was determined by EPR spectroscopy. All EPR spectra were recorded at the same condition as α-Glc-NO. Fe^2+^-(MGD)_2_ (1 mM) was used to trap NO released from CS-NO (0.5 mg/mL in PBS buffer, pH =6). To examine if the reaction was controlled by α-glucosidase, acarbose and heat-denatured α-glucosidase (heated in the boiling water for 5 min) were used as the control group, respectively. NO concentration was calibrated using [2,2,6,6-tetramethylpiperidin-1-yl)oxyl](http://www.lskhsw.com/product/2.html) (TEMPO) as standard.

**

**

Figure s2. EPR spectra of EPR spectra obtained after 3h-incubation of Fe^2+^-(MGD)_2_ (1 mM) with CS-NO (0.5 mg/mL) in the presence of α-glucosidase (i) or heat-denatured α-glucosidase (iii) with the concentration as 0.01 mg/mL; the same as (i) but in the presence of acarbose (1 mM).

1. **PolyMerase Chain Reaction (PCR) analysis**

Table S2. Primer sequences used for Realtime-PCR


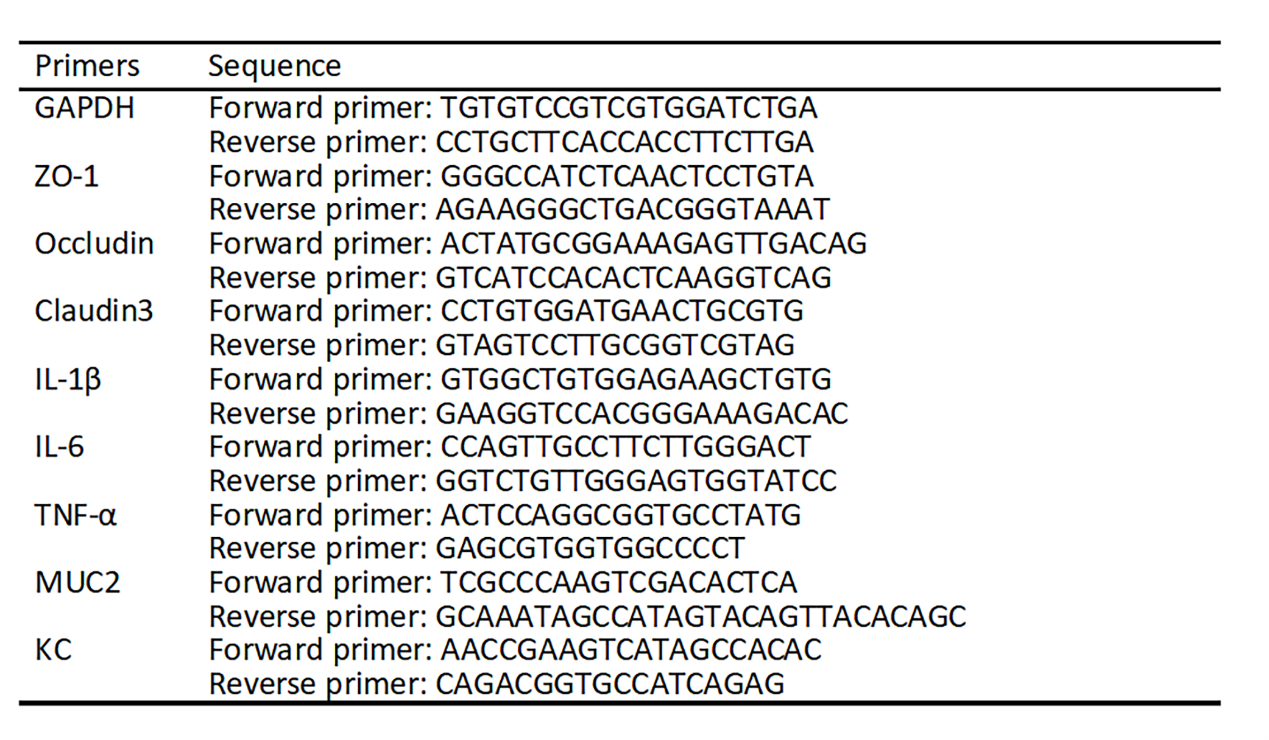


**NMR spectra**


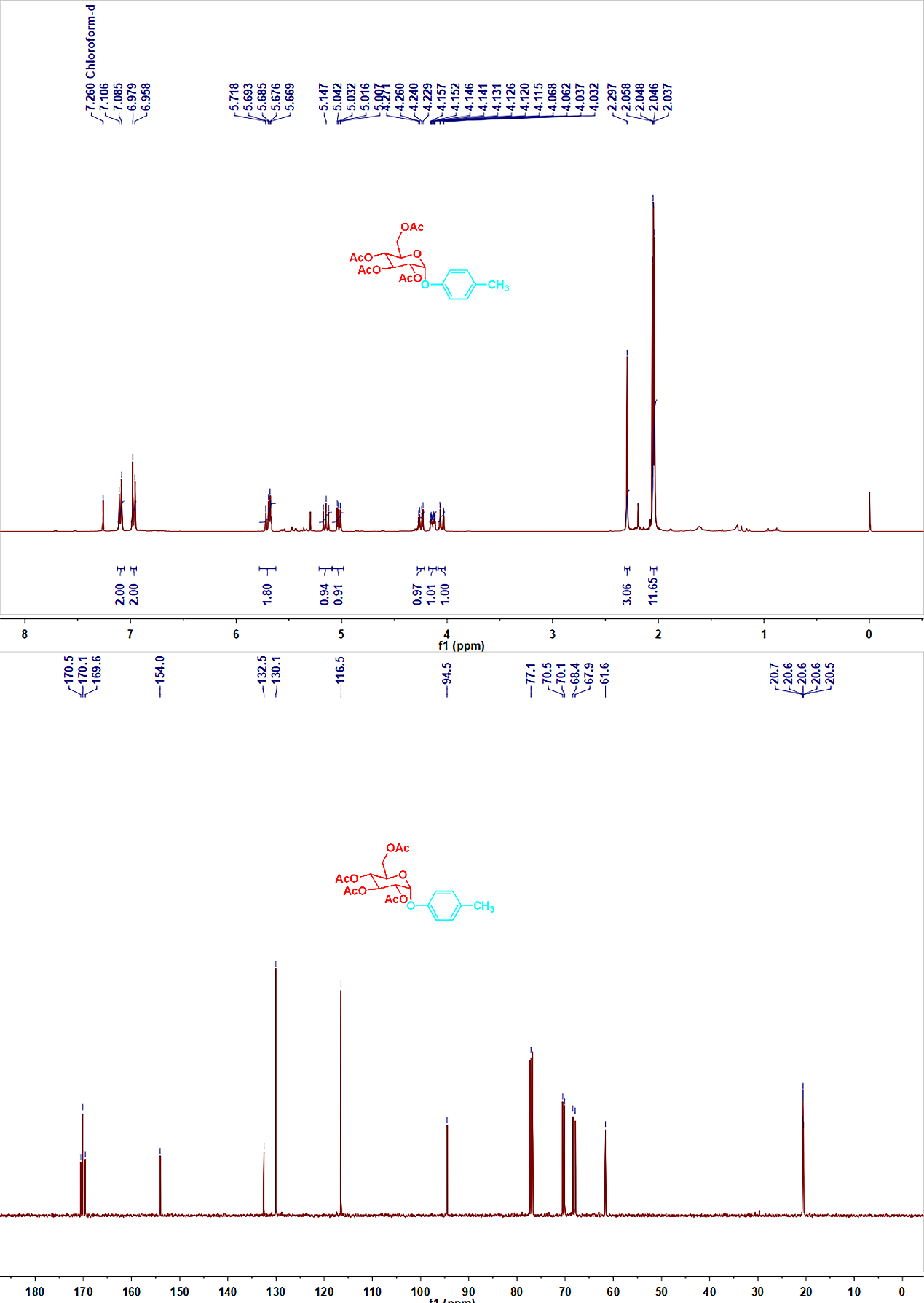


Figure S3 ^1^H NMR and ^13^C NMR spectra of compound **3**


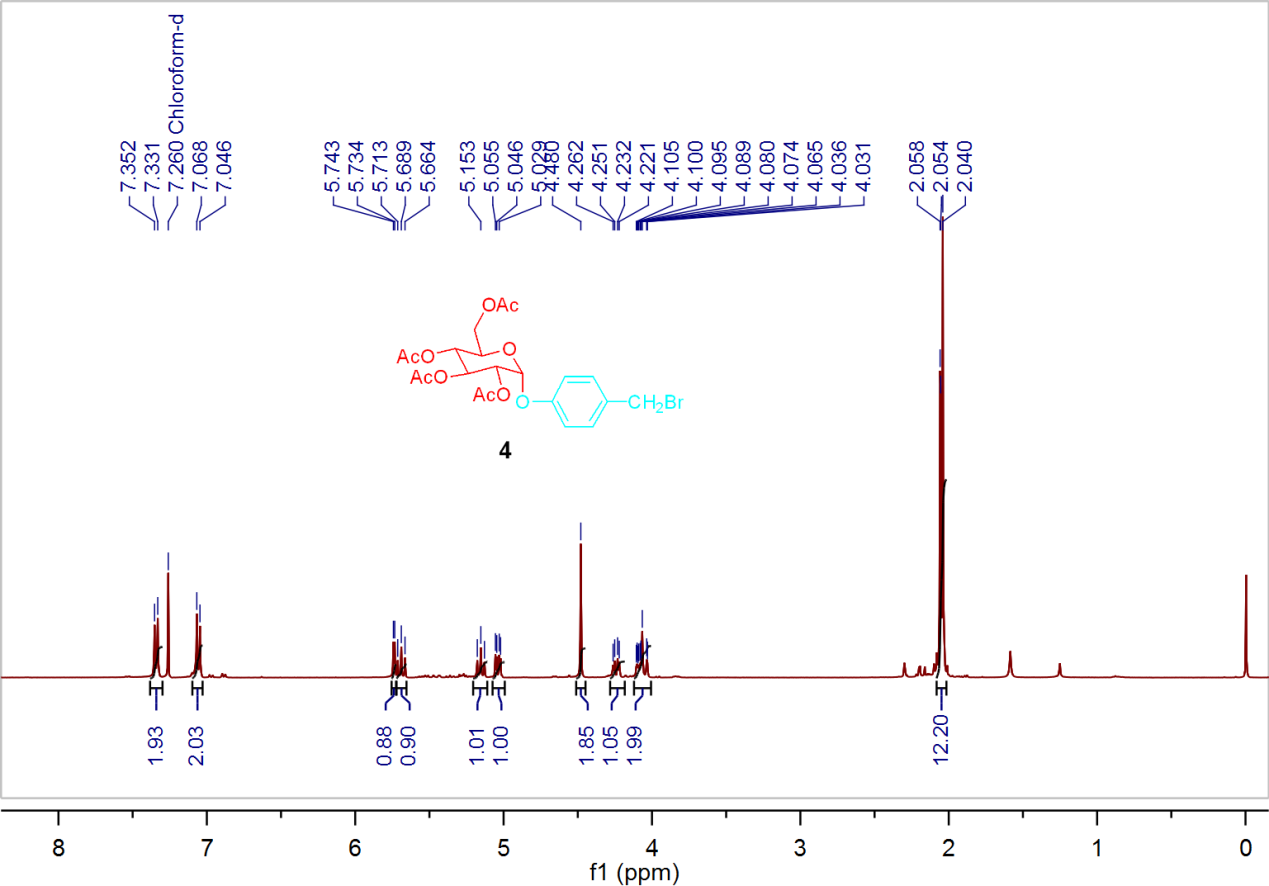

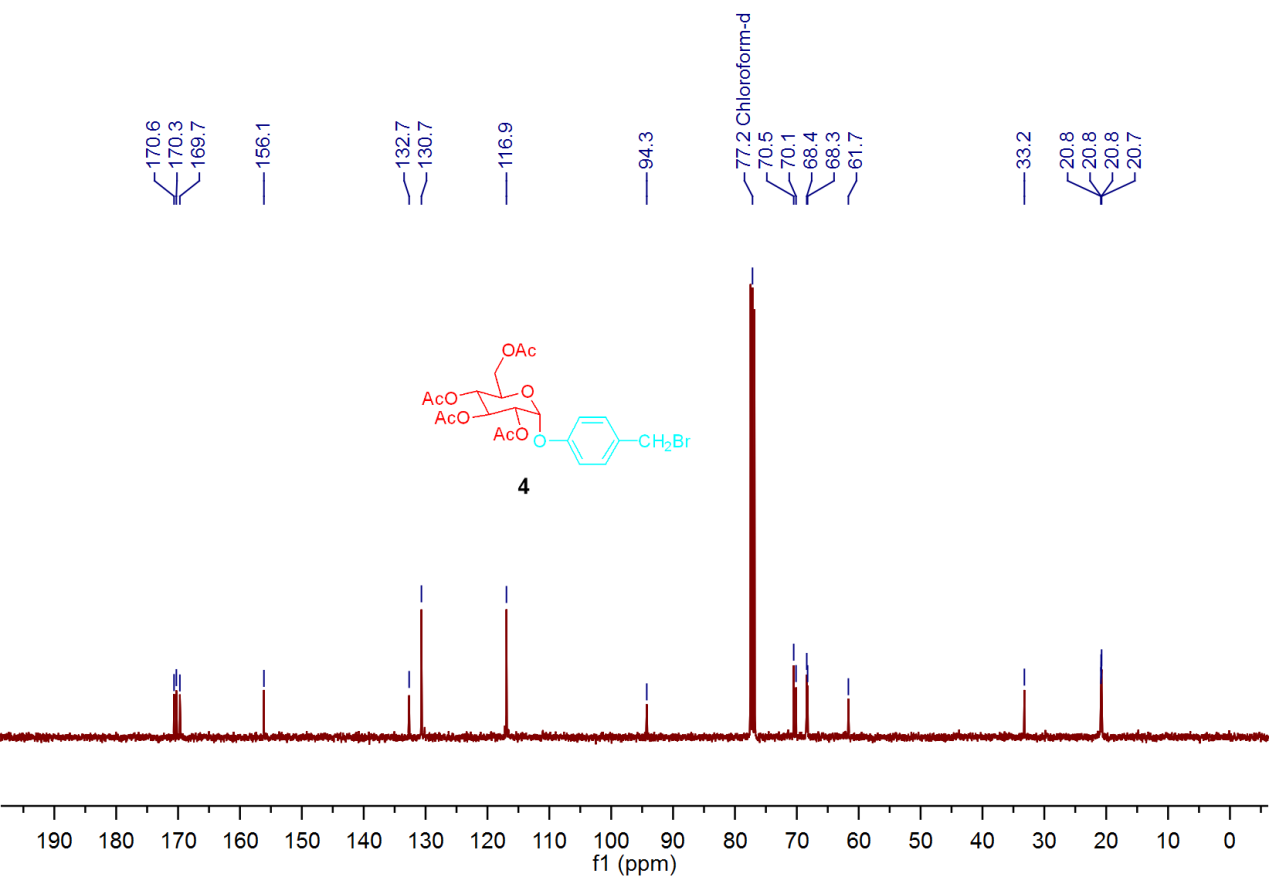


Figure S4 ^1^H NMR and ^13^C NMR spectra of compound **4**


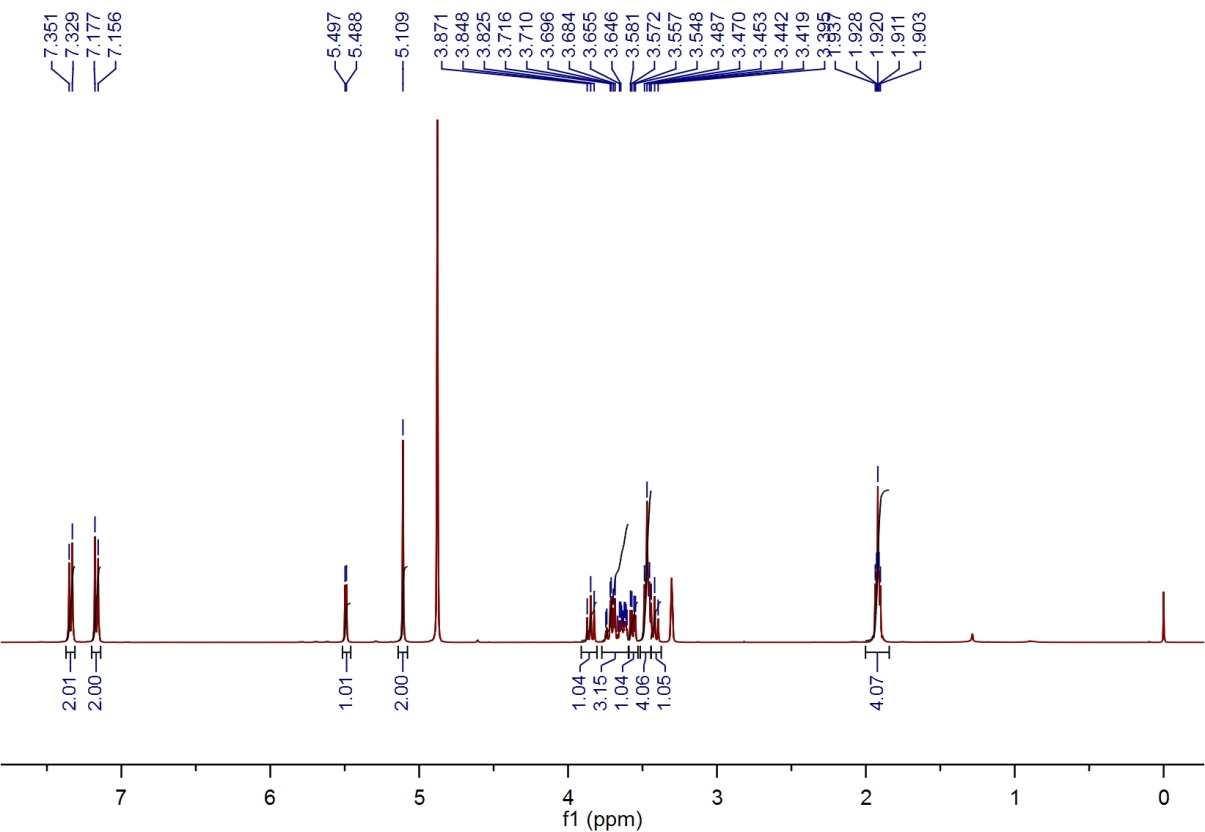

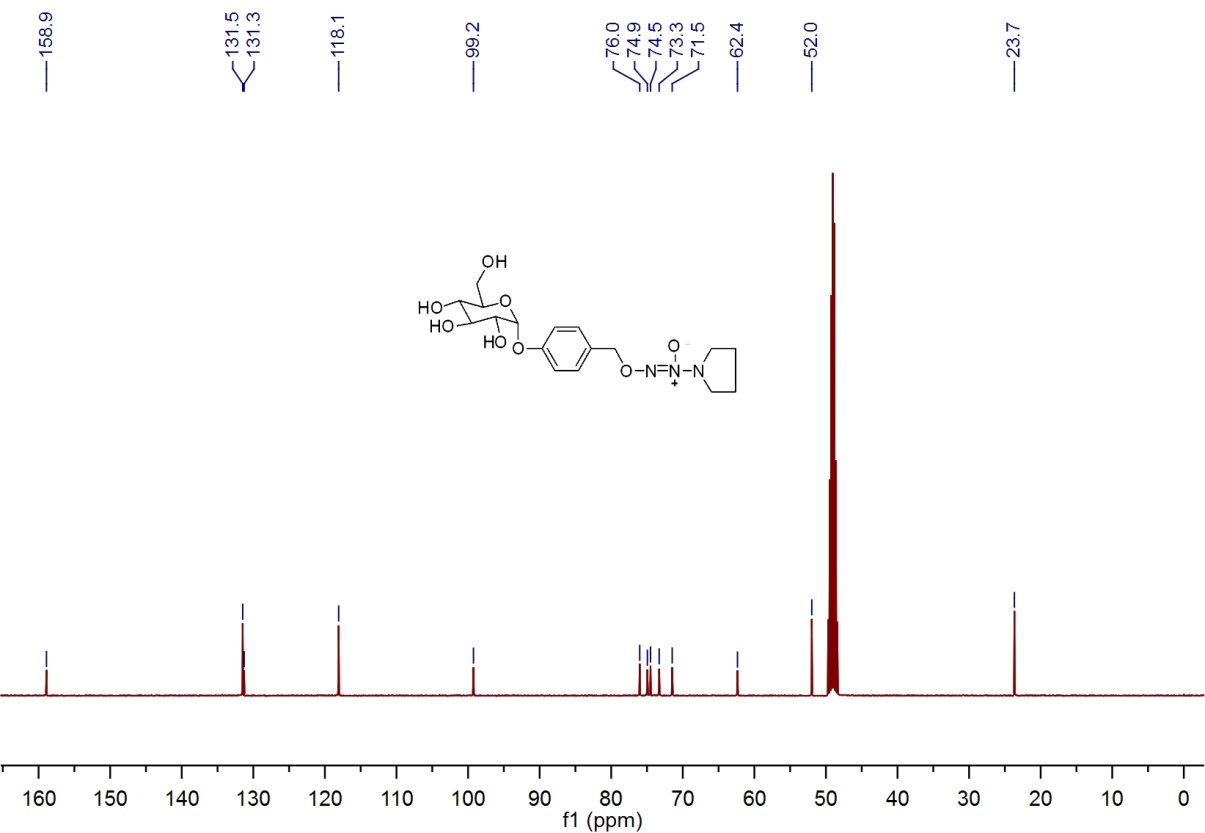


Figure S5 ^1^H NMR and ^13^C NMR spectra of compound **α-Glc-NO**


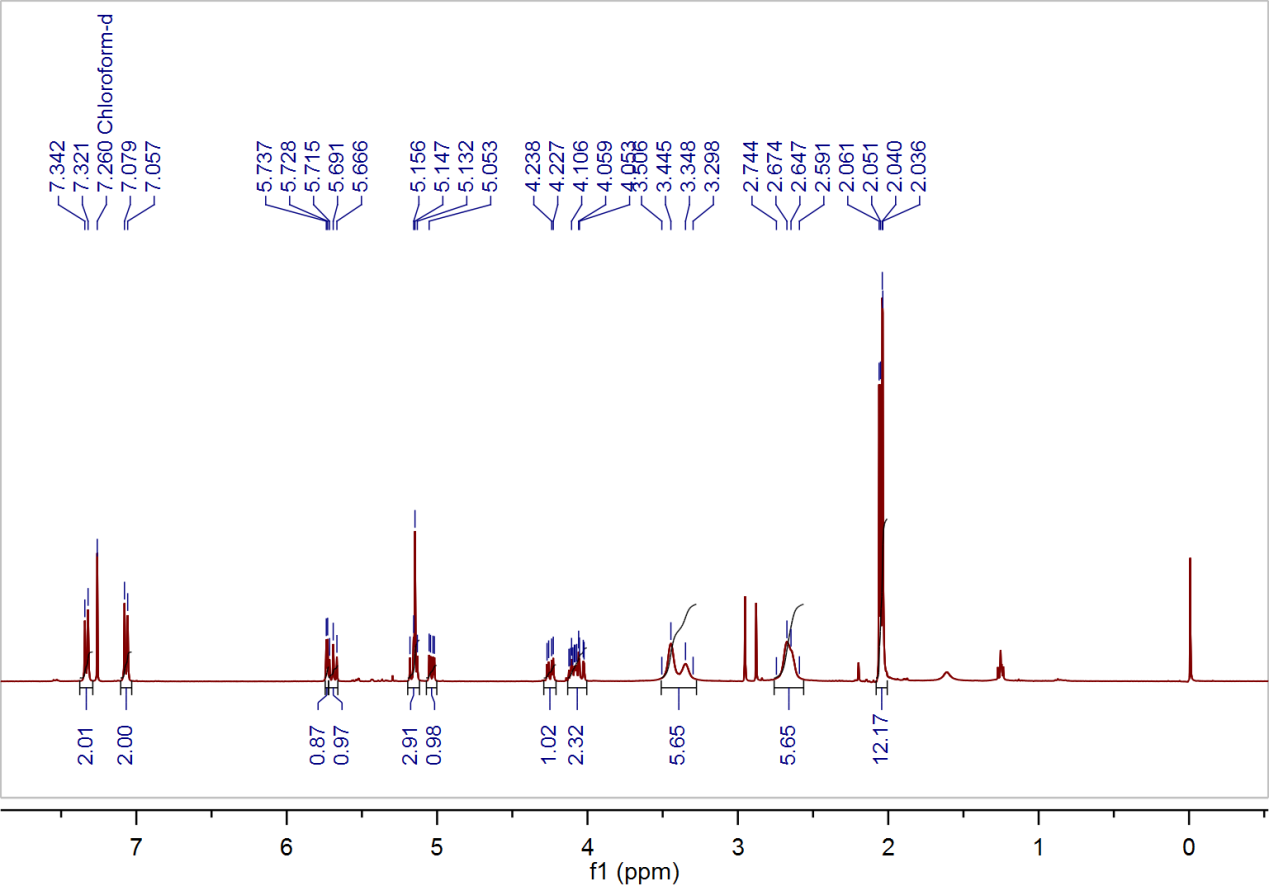

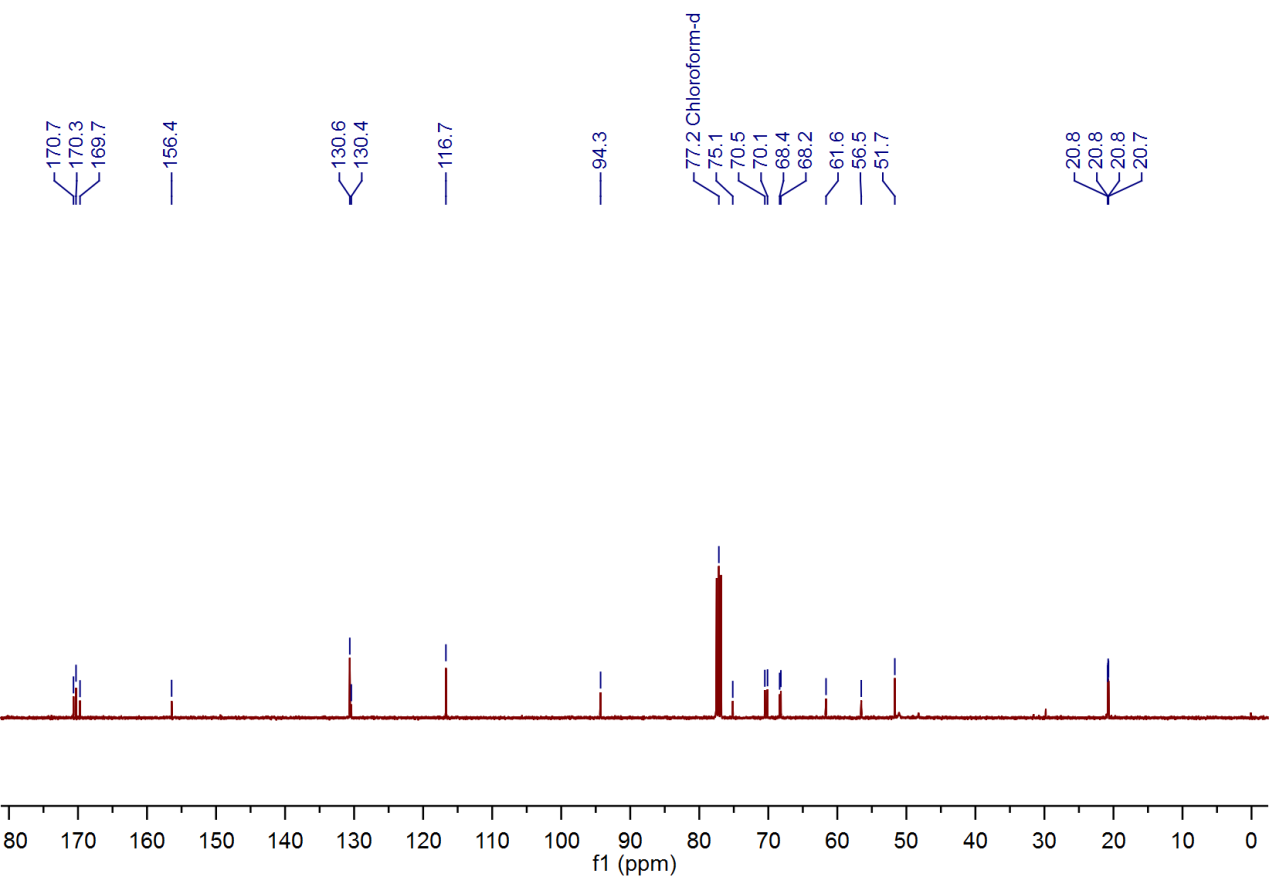

Figure S6 ^1^H NMR and ^13^C NMR spectra of compound **6**


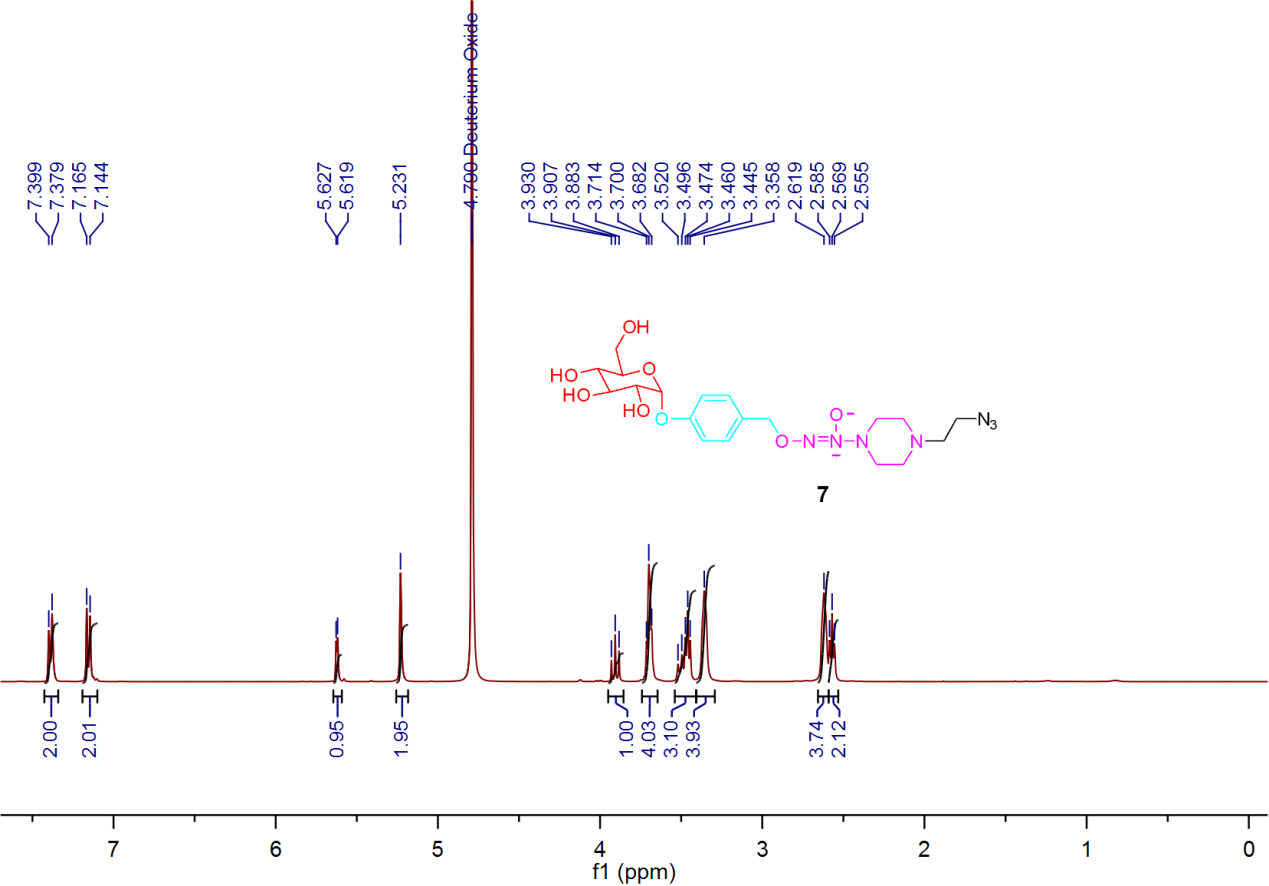

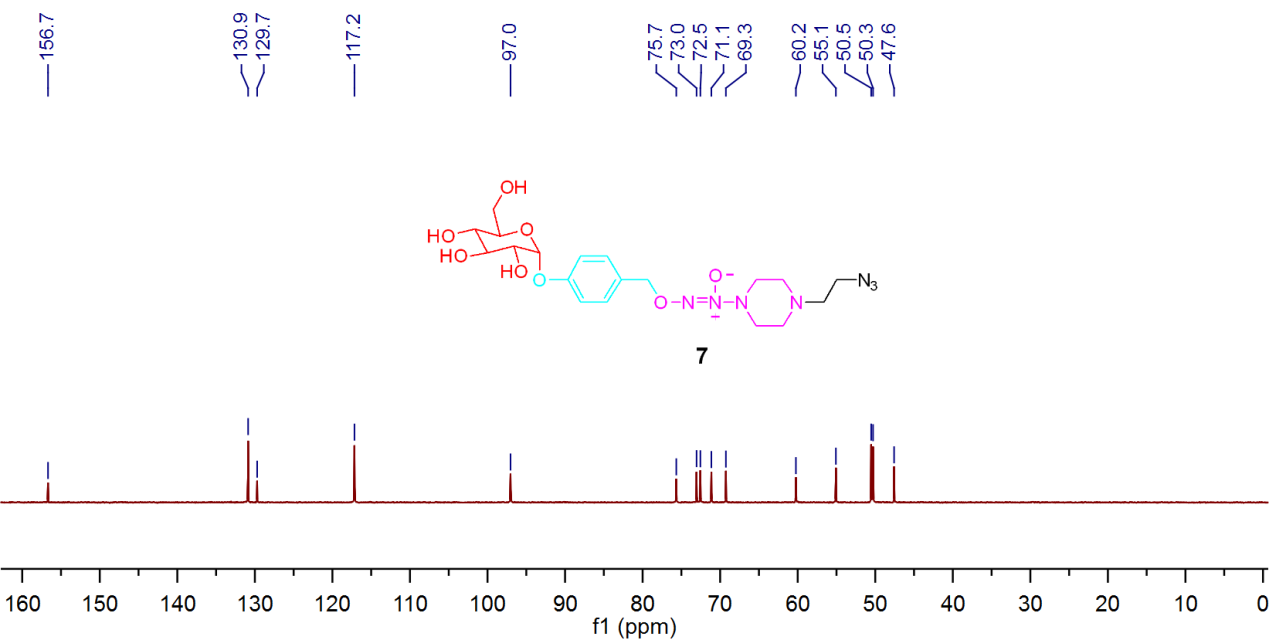


Figure S7 ^1^H NMR and ^13^C NMR spectra of compound **7**


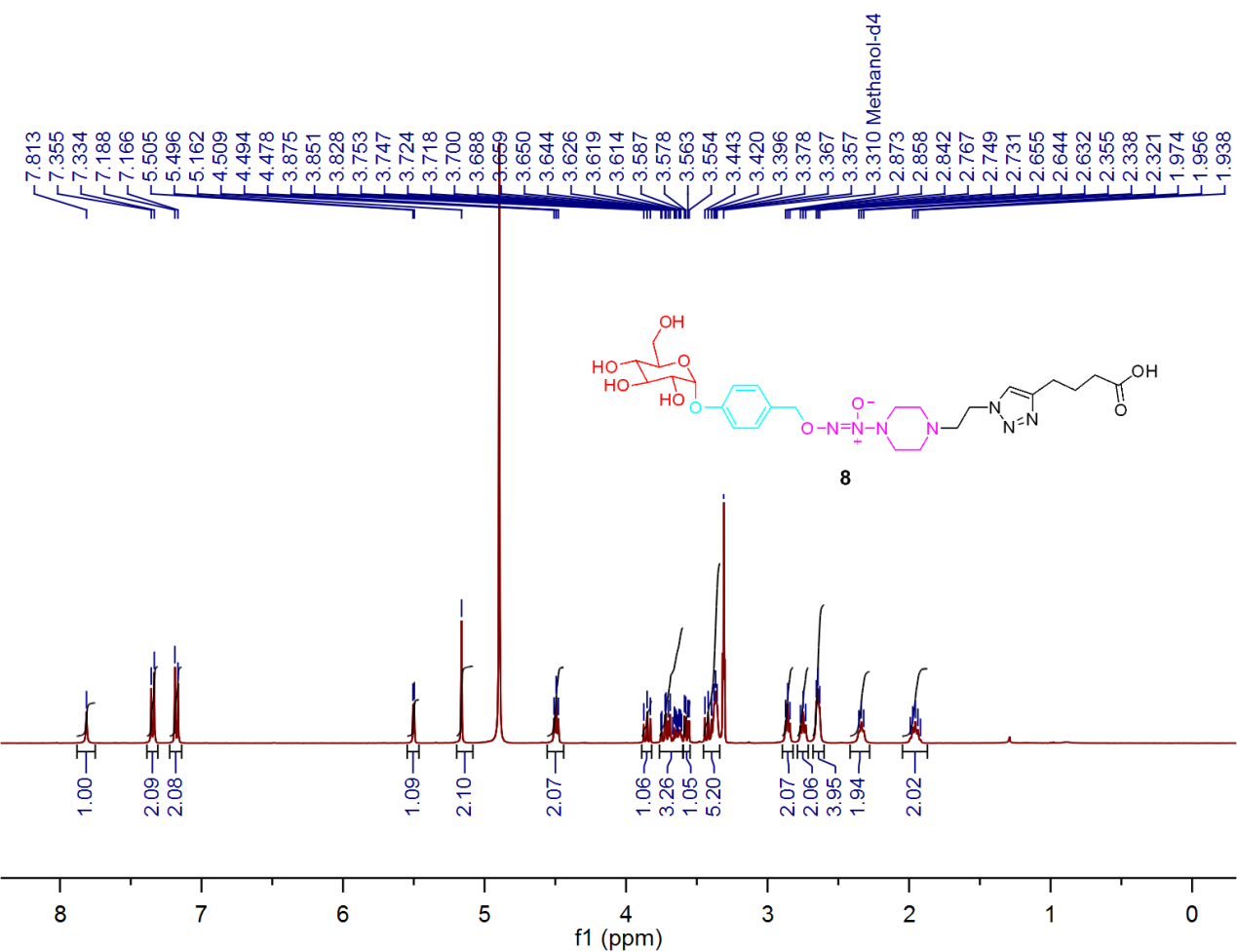


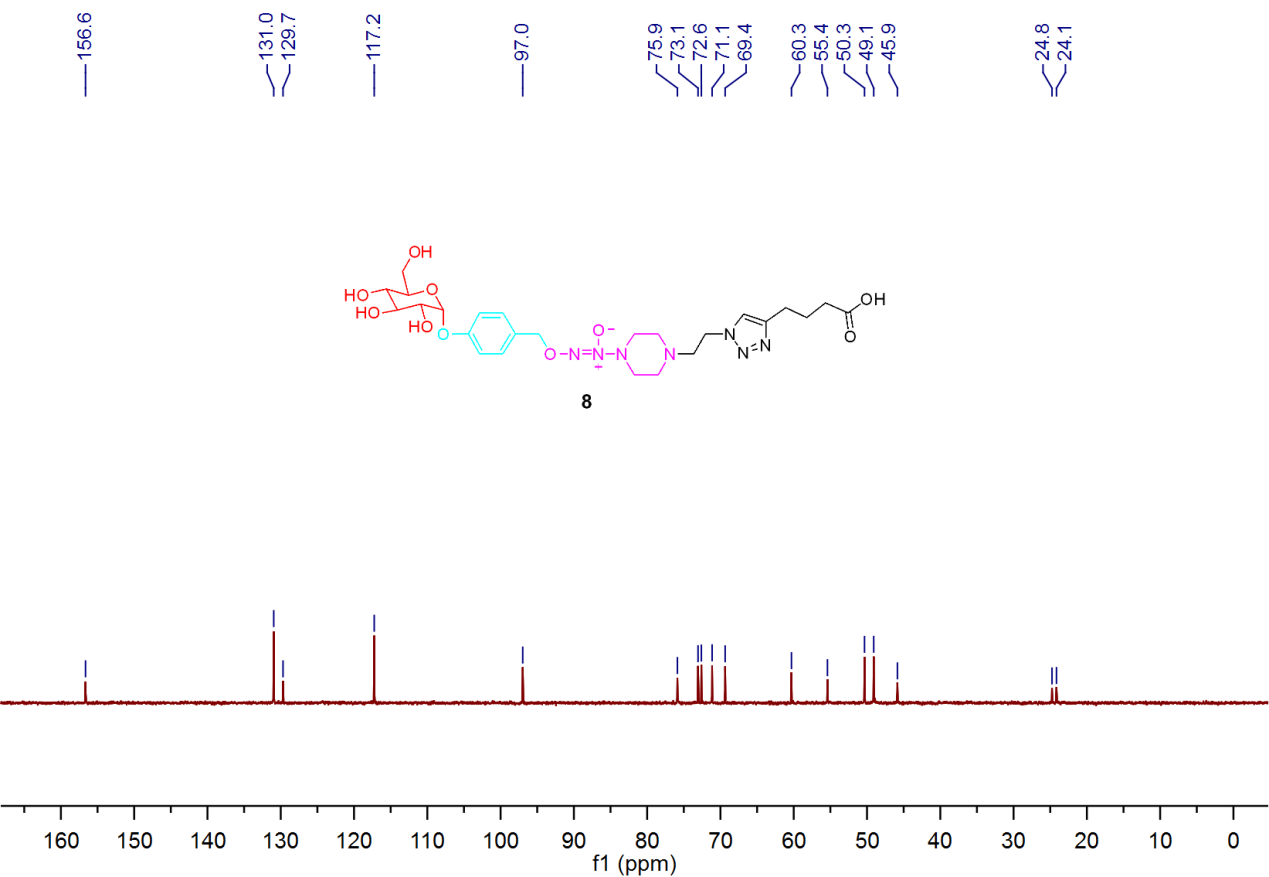


Figure S8 ^1^H NMR and ^13^C NMR spectra of compound **8**


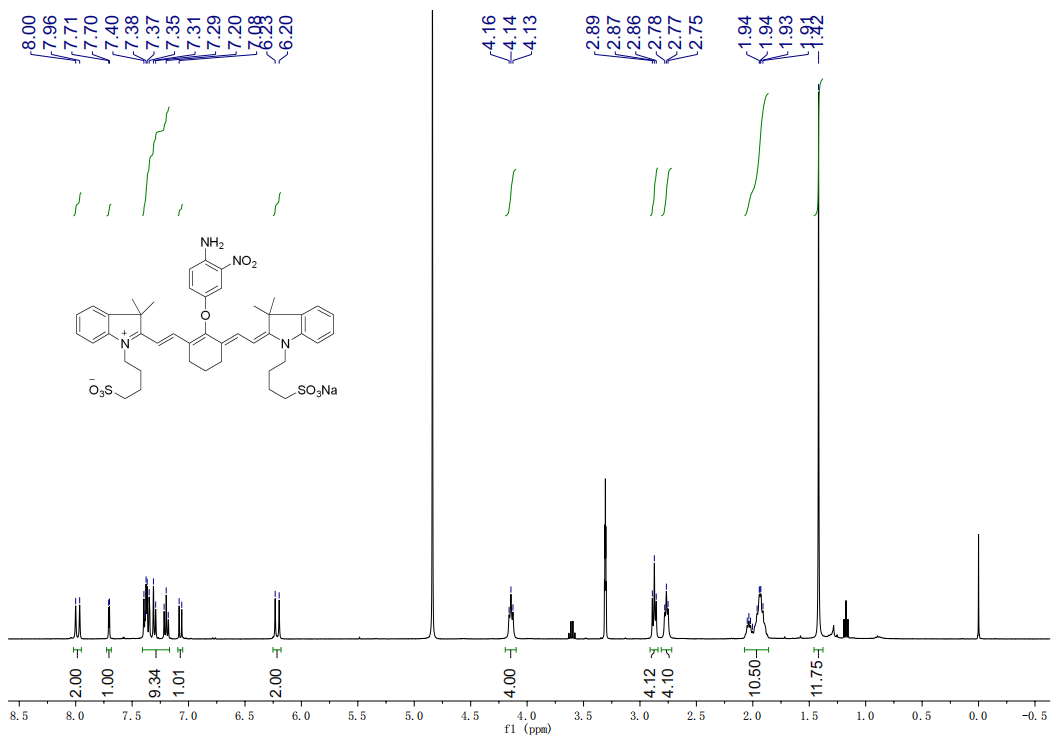


Figure S9 ^1^H NMR spectra of compound **IR-NO_2_**


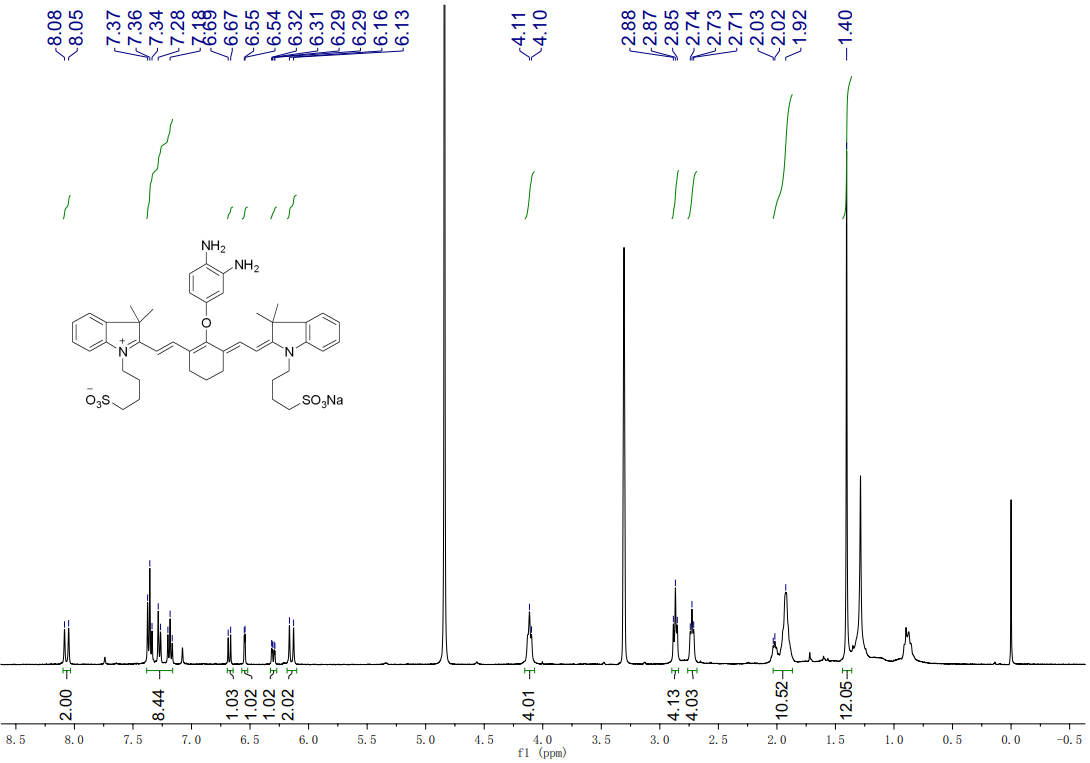


Figure S10 ^1^H NMR spectra of compound **DAC-S,**
